# Harnessing marine polysaccharides for breast cancer therapy: ulvan synergizes with nano-selenium to induce programmed cell death through dual modulation of autophagy and apoptosis

**DOI:** 10.3389/fphar.2026.1968903

**Published:** 2026-10-14

**Authors:** Sara A. Mekkawy, Ahmed M. Abdelmaguid, Manal F. El-Khadragy, Ahmed A. Aly, Mohamed S. Abdelfattah, Mohga S. Abdalla

**Affiliations:** 1 Chemistry Department, Faculty of Science, Capital University (Formerly Helwan University), Cairo, Egypt; 2 Department of Biology, College of Science, Princess Nourah Bint Abdulrahman University, Riyadh, Saudi Arabia; 3 Unit of Scientific Research, Applied College, Qassim University, Buraydah, Saudi Arabia

**Keywords:** apoptosis, breast cancer, Ehrlich solid tumor, molecular docking, oxidative stress

## Abstract

**Background:**

Since breast cancer is one of the biggest worldwide health issues, new and effective treatment approaches are required. Because autophagy control has two context-dependent roles in the development of cancer, it is a prospective therapeutic target. With an emphasis on the regulation of oxidative stress, inflammation, apoptosis, and autophagy, this work assessed the anticancer potential of ulvan, a sulfated polysaccharide produced from Ulva lactuca, and ulvan-loaded nano-selenium in an Ehrlich solid carcinoma model.

**Material:**

ESC-bearing mice were treated with ulvan extract, sodium selenite or ulvan-SeNPs for 14 days. The anticancer activity was assessed through histology, oxidative stress markers, inflammatory mediators, apoptotic proteins and genes expression for autophagy and molecular docking studies.

**Results:**

Molecular docking analysis revealed ulvan-derived saccharides to bind strongly to VEGFR2, CD44 and Toll-like receptors suggesting multi-target action. Ulvan *in vivo* restored antioxidant defenses, reduced oxidative stress and inflammatory markers and induced apoptosis via caspase-3, Bcl-2 and Bax modulation. Ulvan induced controlled autophagy by decreasing mTOR and increasing autophagy related gene expression. Ulvan-SeNPs exhibited enhanced anticancer activity with increased activation of autophagic and apoptotic pathways, as evidenced by tumor shrinkage, reduced angiogenesis and histopathological analysis.

**Conclusion:**

Ulvan is a controlled anti-cancer agent, with a mechanism synergistically enhanced by nano-selenium. These results indicate that ulvan could be a potential natural therapeutic for breast cancer alone or in combination with nano-selenium.

## 1 Introduction

Breast cancer continues to be the most common cancer diagnosed and among the leading causes of death in women worldwide. Breast cancer accounted for one in four cancers and one in six female deaths due to cancer in 2020, with an estimated 2.3 million new cases and 685,000 deaths related to the disease worldwide (Zhang et al., 2025; Perrone et al., 2025). Cancer is still a large burden for many public health systems of different nations, despite improvements in healthcare services (Bizuayehu et al., 2024). According to the World Health Organization, its prevalence is increasing due to a complex relationship of genetic and hormonal factors and exposure to risk in the environment. Even with progress attained in the early detection of cancer and multiple modes of treatment, such as chemotherapy, surgery, radiation therapy, and targeted agents, the incidence of therapeutic resistance, metastasis, and disease recurrence clearly calls for further understanding of the progression of tumors and the establishment of novel treatments (Zafar et al., 2025; Liu et al., 2024).

Autophagy acts as a prominent regulator of homeostasis, as it constitutes a conserved lysosome-dependent degradation pathway that helps cells cope with and remove damaged components under conditions of metabolic stress. The particular context has a marked effect on the role of autophagy within oncology studies (Levy et al., 2017). Point out that “Well-established tumors have been known to exploit autophagy as a prosurvival strategy for continued growth, resistance, and resilience under stressful environments, rather than as an inhibitor for tumorigenesis by protecting genomic integrity, for example, during genomic instability.” The autophagy pathway reveals a complex target for therapy because of its two-faced behavior. Autophagy Function, inhibition, and optimization, and thus direction for appropriate therapy, can be distinguished by precisely assessing autophagic flux, which constitutes the process from autophagy’s induction and clearance, using important autophagy markers that have been recognized for evaluation, such as assessing LC3-II levels and p62/SQSTM1 clearance (Rubinsztein et al., 2012; Khalafiyan et al., 2024).

Recent research has uncovered the promising use of biocompatible nanomaterials and marine polysaccharides as novel anticancer therapies. Selenium is an essential element with proven dose-dependent anticancer activity mediated by pro-oxidative pathways that have been found to induce apoptosis and modulate autophagy. The selenium form at the nanoscale (nano-selenium) enhances bioavailability, decreases toxicity, and induces death of cancer cells by elevating oxidative stress and initiating selective autophagic cell death (cytotoxic autophagy) (Wadhwani et al., 2016).

Ulvan is a sulphated polysaccharide derived from green algae *Ulva lactuca*, which has attracted interest in its various biological activities; these activities include immunomodulatory properties, antioxidant action, and anti-tumor properties. It has been hypothesized that ulvan could have an inhibitory effect on cancer cell proliferation and metastases. This action has been proposed to be associated with regulating cellular autophagy (Kidgell et al., 2019; Wu Y. et al., 2025).

This combination of two drugs signifies a novel and potentially synergistic strategy, as nano-selenium may induce a lethal degree of autophagic flux and oxidative stress. In contrast, ulvan may improve immune function and further interfere with pathways that facilitate tumour survival. However, the combined impact of these factors on the regulation of autophagic flux in a living tumor microenvironment remains unexamined.

Although individual studies indicate anticancer activity for nano-selenium and ulvan, there is a lack of research on their combined effects on breast cancer. Furthermore, their specific influence on autophagic flux dynamics has not been systematically characterized in an appropriate *in vivo* Erlich model to address this gap effectively.

## 2 Materials and methods

### 2.1 *Ulva lactuca* seaweed collection

The *U. lactuca* seaweed was gathered in July 2023 at a depth of 0.5 m from Miami Beach, Alexandria, Egypt (31°15'43.0”N 29°58'57.5” E). Fresh plants, after getting rid of epiphytes and subsequently rinsed with seawater, were placed in plastic bags. On arriving at the lab, the seaweed was rewashed using distilled water and left to dry in the air for 72 h at 35 °C. Following the drying process, the alga was ground to a homogeneous powder using a mechanical grinder for 5 minutes. It was then packed in an airtight bag and stored in a dry and dark place at room temperature (25 °C) until needed.

### 2.2 Sulphated polysaccharides extraction

Various extractions are conducted by heating 10 g of algal powder in 200 mL of HCl solution (pH 1.5 and pH 2) while stirring at 250 rpm in a jacketed stainless steel reactor flask with a thermostatic bath, utilizing a discontinuous process at temperatures of 80 °C and 90 °C for 3 hours. After extraction, the suspension was allowed to cool to room temperature before filtration through cheesecloth. The filtrate was centrifuged for 20 min at 10,000 rpm at ambient temperature to eliminate solid particles. The supernatant was then filtered again with Whatman filter paper No. 1 to remove the impurities. The obtained filtrate was called the extract juice. One volume of the extracted juice was mixed with four volumes of 96% V/V ethanol for the precipitation of ulvan, and the mixture was kept in a refrigerator for 24 hours. Centrifugation was used to separate the alcohol precipitate from the supernatants for 20 min at 10 °C and 5,000 rpm. After three rounds of washing with 50%, 75%, and 100% ethanol, the alcohol precipitate was subjected to centrifugation for 10 min at 10 °C and 5,000 rpm. Ultimately, it was desiccated to a uniform weight at 40 °C in a vacuum oven before being finely pulverized in a centrifugal mill (Mekkawy et al., 2025; Auta, 2017; Ibraheem and Babatunde, 2019). The functional groups of the isolated ulvan were verified using a Fourier-transform infrared spectrophotometer (FT-IR) using a PerkinElmer ATR sample base plate (DIAMOND).

### 2.3 Preparation of nano-selenium

Ulvan was used as a reducing agent to reduce sodium selenite while generating nano-selenium. After preparation of 0.1 M sodium selenite, ulvan extract was added gradually with continuous stirring for 24 h. A very small amount of 1% ascorbic acid can be prepared and progressively added to the mixture until a red or red-orange colour appears as an indication of the formation of nano-selenium (Mekkawy et al., 2026). To verify the nano production, Zeta sizer, FTIR, TEM, and potential analyses were conducted.

### 2.4 Molecular docking

#### 2.4.1 Protein preparation (multi-target approach)

The three-dimensional crystal structures of seven key target proteins associated with inflammation, angiogenesis, immune modulation, tumor progression, and cell survival in the Ehrlich tumor model were retrieved from the Protein Data Bank (PDB). The selection of these targets was based on their reported involvement in biological processes that are potentially relevant to the proposed antitumor and immunomodulatory activities of ulvan. The selected targets represent complementary biological pathways, including innate immune signaling, cell adhesion and extracellular matrix interactions, angiogenic signaling, tumor progression, and tumor-cell survival. The selected protein targets, their biological significance, relevance to the Ehrlich tumor model, PDB accession numbers, and the corresponding molecular docking search-space parameters are summarized in Table 1.

**TABLE 1:** Selected protein targets and molecular docking search-space parameters for the ulvan-derived trisaccharide fragment in the Ehrlich tumor model.

No.	Protein target	Main pathway/biological importance	Relevance to the ehrlich tumor model	PDB ID	Center X	Center Y	Center Z	Size X (Å)	Size Y (Å)	Size Z (Å)
1	Toll-like receptor 4 (TLR4)	Innate immunity and immunomodulation	Involved in tumor–immune interactions and inflammatory signaling	3FXI	11.961	22.523	−12.539	30	30	30
2	CD44 (hyaluronan receptor)	Cell adhesion and migration	Associated with tumor-cell adhesion, extracellular matrix interactions, and tumor progression	1UUH	−4.306	−8.521	0.352	30	30	30
3	Vascular endothelial growth factor A (VEGF-A)	Angiogenesis	Important mediator of tumor-associated angiogenic signaling	1VPF	−8.411	−3.604	48.156	30	30	30
4	Galectin-3 (LGALS3)	Immune escape and tumor survival	Associated with tumor progression, cell survival, and resistance to apoptosis	1A3K	4.566	7.356	−14.501	30	30	30
5	Fibroblast growth factor 2 (FGF2)	Angiogenesis and proliferation	Contributes to tumor growth and angiogenic processes	1BFC	−6.968	−5.055	40.962	30	30	30
6	Toll-like receptor 2 (TLR2)	Innate immune signaling	Involved in innate immune responses and polysaccharide-mediated immunomodulation	2Z7X	−17.253	−13.235	13.383	30	30	30
7	Vascular endothelial growth factor receptor 2 (VEGFR2/KDR)	Angiogenic signaling	Major VEGF receptor involved in tumor vascularization	3WZE	26.138	23.098	35.598	30	30	30

The protein structures were selected based on the availability of experimentally determined three-dimensional structures and structurally characterized functional or ligand-binding regions suitable for molecular docking analysis. The final target panel comprised Toll-Like Receptor 4 (TLR4; PDB ID: 3FXI), CD44 (PDB ID: 1UUH), Vascular Endothelial Growth Factor A (VEGF-A; PDB ID: 1VPF), Galectin-3 (LGALS3; PDB ID: 1A3K), Fibroblast Growth Factor 2 (FGF2; PDB ID: 1BFC), Toll-Like Receptor 2 (TLR2; PDB ID: 2Z7X), and Vascular Endothelial Growth Factor Receptor 2 (VEGFR2/KDR; PDB ID: 3WZE). The inclusion of these targets enabled a multi-target docking strategy to investigate the potential interactions of the ulvan-derived trisaccharide fragment with proteins representing different molecular pathways implicated in tumor development and progression.

Protein preparation was performed using AutoDock Tools (ADT), version 1.5.7 (Eltanany et al., 2026). A standardized preparation protocol was applied to all seven target proteins to ensure consistency among the docking calculations. Crystallographic water molecules were removed from the protein structures, and co-crystallized ligands were removed where appropriate, while the structural information provided by the original ligands was considered when identifying the relevant binding regions. Polar hydrogen atoms were subsequently added, Kollman partial atomic charges were assigned, and non-polar hydrogen atoms were merged. The prepared protein structures were then converted and saved in PDBQT format, which is required for molecular docking calculations using AutoDock Vina (Trott and Olson, 2010; Zakaria et al., 2026).

Molecular docking calculations were subsequently performed using AutoDock Vina. The docking search space was defined individually for each target protein according to the selected binding region. A uniform search-space size was applied to the selected targets, while the center coordinates were adjusted according to the corresponding protein structure and binding region. The complete grid-box dimensions and center coordinates used for each target protein are provided in Table 1. This approach ensured that the same defined search-space dimensions were consistently applied while allowing the grid center to be appropriately positioned for each individual protein target.

The optimized trisaccharide-based ulvan-derived ligand described in Section 2.4.2 was used as the single ligand model for all molecular docking simulations. The three selected monosaccharide components were combined to generate this trisaccharide-based ulvan fragment before docking, rather than being treated as separate ligands. For each protein target, the ligand was positioned within the corresponding predefined docking search space, and AutoDock Vina was used to identify and rank the energetically favorable binding poses. The best-ranked docking pose for each target was subsequently selected for interaction analysis and for the calculation of the predicted binding affinity and inhibition constant (Ki). The docking results were then compared across the selected targets to evaluate the relative interaction potential of the ulvan-derived trisaccharide fragment with proteins involved in different tumor-associated pathways.

The docking protocol and search-space parameters were kept consistent across the target panel to facilitate comparative evaluation of the predicted ligand–protein interactions. The resulting binding modes were visualized and analyzed based on the predicted binding energies and the intermolecular interactions established between the ulvan-derived ligand and the corresponding amino acid residues within the predicted binding sites.

#### 2.4.2 The ligand preparation

The ligand preparation was based on selected monosaccharide components reported in the structural composition of ulvan, a sulfated heteropolysaccharide mainly composed of rhamnose, uronic acids, and xylose residues (Kidgell et al., 2019; Utami et al., 2023). In the present study, three selected ulvan-derived monosaccharide components, namely glucuronic acid, rhamnose-sulfate, and xylulose, were used as building blocks to construct a single trisaccharide-based ulvan-derived ligand. These three monosaccharide units were combined to generate a defined oligosaccharide fragment representing a simplified structural motif of the ulvan polysaccharide. The resulting trisaccharide structure was assembled using a glycan-building tool and subsequently subjected to geometry optimization and energy minimization using Avogadro with the MMFF94 force field to obtain a stable, low-energy three-dimensional conformation. The optimized trisaccharide-based ulvan fragment was then used as the single ligand model for all molecular docking simulations against the selected protein targets.

### 2.5 *In vivo* model

We conducted our experiment on 40 female BALB/c mice (8–9 weeks old, weighing 31–35 g), maintained under standard temperature conditions with a regular 12-h light/12-h dark cycle. Prior to the experiment, the animals were allowed to acclimatize to the laboratory conditions. During the study period, they were not subjected to any restrictions on activity or loading stress. The Research Ethics Committee of the Faculty of Science, Capital University, approved the experimental protocol under Approval No. HU-IACUC/Z/AES0524-1. The mice were randomly assigned to five treatment groups (n = 8/group) using a computer-generated randomization sequence (Random Allocation Software version 2.0). During the treatment period one animal per group was excluded from biochemical and molecular analyzes because of technical problems in tissue homogenization (sample quality was compromised). All biochemical and molecular data are expressed as mean ± SEM with n = 7 per group. Histopathological and immunohistochemical analysis was performed on the same n = 7 animals per group.

Ehrlich ascites carcinoma (EAC) cells (1 × 10^6^ cells) obtained from the National Cancer Institute (NCI), Cairo University, were intraperitoneally injected into female BALB/c mice. On the seventh day following the injection, the ascitic fluid was aspirated. The Ehrlich cells were then washed twice and finally resuspended in RPMI-1640 medium. EAC cells were authenticated based on morphological criteria, and cell viability was assessed using trypan blue dye exclusion before injection. All experimental groups received an intramuscular injection of 0.2 mL containing 1 × 10^6^ viable Ehrlich cells into the thigh of the right hind leg and were left for 7 days without treatment to allow solid tumor development. The animals were then divided and treated as follows:

Control group: The mice in this group received no treatment throughout the experimental period.

Na-selenite group: Mice in this group were administered sodium selenite orally at a dose of 2.5 mg/kg body weight once daily for 14 days (Sager, 2006).

Ulvan group: Mice in this group were administered ulvan orally at a dose of 100 mg/kg body weight once daily for 14 days, based on previously reported studies (Pradhan et al., 2023).

Ulvan–NanoSe group: Mice in this group were administered ulvan-mediated selenium nanoparticles (Ulvan-SeNPs) orally at a dose of 0.5 mg/kg body weight once daily for 14 days, based on our previously reported study (Mekkawy et al., 2026).

Cisplatin (CP) group: Mice in this group were administered cisplatin intraperitoneally (i.p.) at a dose of 5 mg/kg body weight on days 1, 4, and 8 of the treatment period (three doses in total), based on established protocols in the Ehrlich solid carcinoma model (Eissa et al., 2025).

At the end of the experiment, the mice were euthanized with an overdose of pentobarbital (300 mg/kg body weight). The tumor in the right hind leg was removed and measured. A segment of the muscle tissue containing Ehrlich solid carcinoma (ESC) was fixed in 10% buffered formalin for morphological and immunohistochemical analyses. A separate fraction was homogenized in ice-cold sodium–potassium phosphate buffer and subsequently preserved at −80 °C until further biochemical analyses.

### 2.6 Assessment of NaSe, ulvan, NanoSe, and CP effect on body weight, tumor weight, tumor volume, and tumor growth inhibition index

An electronic digital dual-end calliper (from Switzerland) was utilized for the measurement of the size of the right thigh biweekly, starting on day 7 following the formation of tumors. Changes in tumor volume were calculated using the following formula, described by Goto et al. (Goto et al., 2000):

$$\text{TV }\left(\text{mm}3\right)=\left[\left(W2\times L\right)/2\right]$$

where L is the length of the tumor, and W is the width of the minor tumor. In addition, the tumor growth inhibition index (T/C%) was calculated using the following formula, which was quoted by Hather et al. (Hather et al., 2014):

$$T/C\%=\left[1-\left(\text{mean volume of treated tumors}\right)/\left(\text{mean volume of control tumors}\right)\right]\times 100\%.$$

### 2.7 Oxidative stress and antioxidant biomarkers in the ESC tissue

To assess the level of oxidative stress in the ESC tissue, biochemical analysis was performed on muscle homogenates that were taken at the end of the experimental period.

Lipid peroxidation (LPO) and nitric oxide (NO) were evaluated by the described method by Ohkawa et al. (Ohkawa et al., 1979) and Green et al. (Green et al., 1982), respectively. The activities of superoxide dismutase (SOD), catalase (CAT), glutathione peroxidase (GPx), and glutathione reductase (GRd) were analyzed according to the previously described methods (Fac et al., 1998; Paglia and Valentine, 1967; Luck, 1963; Sun et al., 1988), respectively. Also, the glutathione content was approximately dependent on the method described by Ellman (Ellman, 1959). The concentration of protein in the homogenates was estimated through the described method by Bradford (Bradford, 1976), and the data were expressed in mg protein^−1^.

### 2.8 Biomarkers for ESC tissue inflammation and apoptosis

Levels of the pro-inflammatory cytokines tumor necrosis factor-α (TNF-α), NF-κB p65, and interleukin-1β (IL-1β) were evaluated using ELISA kits (PeproTech, Rocky Hill, NJ, USA) according to the manufacturer’s instructions.

### 2.9 Apoptotic protein markers

Bcl-2 and Bax levels were assessed using ELISA kits (Elabscience, Houston, TX, USA) following the manufacturer’s guidelines. Caspase-3 activity was assessed using a colorimetric assay kit obtained from Sigma-Aldrich (St. Louis, MO, USA).

### 2.10 Autophagy-related gene expression studies

The total RNA was extracted from the ESC Muscle Tissue using the RNeasy Mini Kit purchased from Sigma-Aldrich (Louis, MO, USA, Cat. No. 74104) according to the manufacturer’s protocol. The total RNA was reverse transcribed immediately using a RevertAid Reverse Transcriptase (Thermo Fisher, Cat. No: EP0441). Real-time PCR was performed using the Quantitect SYBR green PCR kit (Cat. No. 204141) to amplify the PCR products on the Stratagene MX3005P instrument. The primers used in the PCR analysis were purchased from Sigma-Aldrich (Louis, MO, USA). Primer sequences have been designed in the current study and are listed in Table 2. The results were expressed as the relative expression ratio between the targeted gene (mTOR, Beclin-1, ATG-5, ATG-7) and the glyceraldehyde-3-phosphate dehydrogenase (GAPDH) reference gene. The relative gene expression was determined using the 2^−ΔΔCT^ method.

**TABLE 2:** Primer sequences of targeted genes (MTOR, Beclin-1, ATG-5, ATG-7, and GAPDH).

Gene	Primer sequence (5'-3')	References
Mtor	GCA​TAA​CAG​ATC​CTG​ACC​CTG​AT	Designed in the current study
	TTC​AGA​GCC​ACA​AAC​AGA​GC	
Beclin-1	GTG​GAA​AAG​AAC​CGC​AAG​GT	
	CCC​GCT​GGT​ACT​GAG​CTT​C	
ATG-5	TGC​GGT​TGA​GGC​TCA​CTT​TA	
	CAA​AAC​TGG​TCA​AAT​CAT​TCT​GC	
ATG-7	GAG​GAG​GAC​TTT​GGT​TGC​AGG	
	GGC​TAC​TGT​TCT​TAC​CAG​CCT​GT	
GAPDH	AAC​CCT​TAA​GAG​GGA​TGC​TGC	
	TCT​ACG​GGA​CGA​GGA​AAC​AC	

### 2.11 Histopathological and immunohistochemical examinations

The ESC muscle tissue specimens were preserved in 10% neutral-buffered formalin at 24 °C for a duration of 24 h. Following fixation, the samples underwent dehydration with ethyl alcohol, were cleared in xylene, and subsequently embedded in paraffin. Serial sections with a thickness of 5 μm were deparaffinized and stained using hematoxylin and eosin (H&E) or subjected to immunohistochemical examination for VEGF, NF-κB, and LC-3. The ESC muscle tissue sections were examined using a light microscope (Kontron Electronic Imaging System KS300, Zeiss, Germany) at a magnification of 400×.

### 2.12 Statistical analysis

All acquired data were examined utilizing a one-way ANOVA test, followed by Duncan’s *post hoc* test. A *p-*value of less than 0.05 was established to ascertain statistical significance. The examined data were presented as the mean ± standard error of the mean (SEM).

## 3 Results

### 3.1 Characterization of ulvan and ulvan nano-selenium

The FT-IR spectroscopy analysis (Figure 1) was employed to characterize the functional groups of ulvan and the ulvan-SeNPs composite. The spectra for both Ulvan (black line) and ulvan-SeNPs (red line) exhibited a broad band centered around 3,373 cm^-1^, signifying the presence of hydroxyl groups (O-H stretch) in the ulvans. The bands associated with the asymmetric and symmetric stretching vibrations of the carboxylate (COO^−^) groups are observed between 1,621 cm^-1^ and 1,423 cm^-1^ in both samples, attributable to the uronic acid residues within the ulvan structure. The prominent band at 1,031 cm^-1^ is ascribed to the stretching vibration of the glycosidic bonds (C-O-C stretch) in the ulvan polysaccharide. Both Ulvan and Ulvan-SeNPs exhibited a distinctive peak about 844 cm^-1^, indicative of the bending vibrations of C-O-S bonds in sulfate groups. This band is identified as a distinctive marker of ulvan.

**FIGURE 1:**
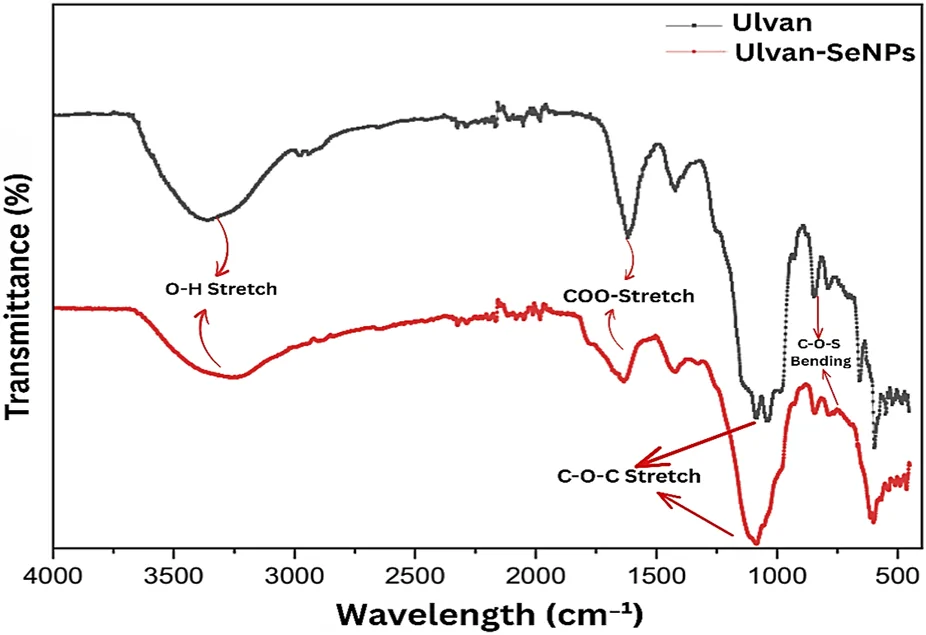
Functional group characterization of ulvan and ulvan-SeNPs by FT-IR spectroscopy.

The physical characteristics of the produced ulvan-conjugated selenium nanoparticles were validated using Dynamic Light Scattering (DLS) and Zeta potential analysis (Figure 2). The DLS analysis (Figure 2A) demonstrated a tight, homogenous size distribution for the ulvan-SeNPs, with a mean hydrodynamic diameter of 48.5 ± 2.1 nm and a low polydispersity index (PDI) of 0.21, signifying exceptional colloidal stability. The Zeta potential study (Figure 2B) indicated that the ulvan-SeNPs exhibit a significant negative surface charge of −31.7 ± 1.5 mV. These measures validate the effective biogenic production of suitably sized nanoparticles and their considerable surface stability in solution.

**FIGURE 2:**
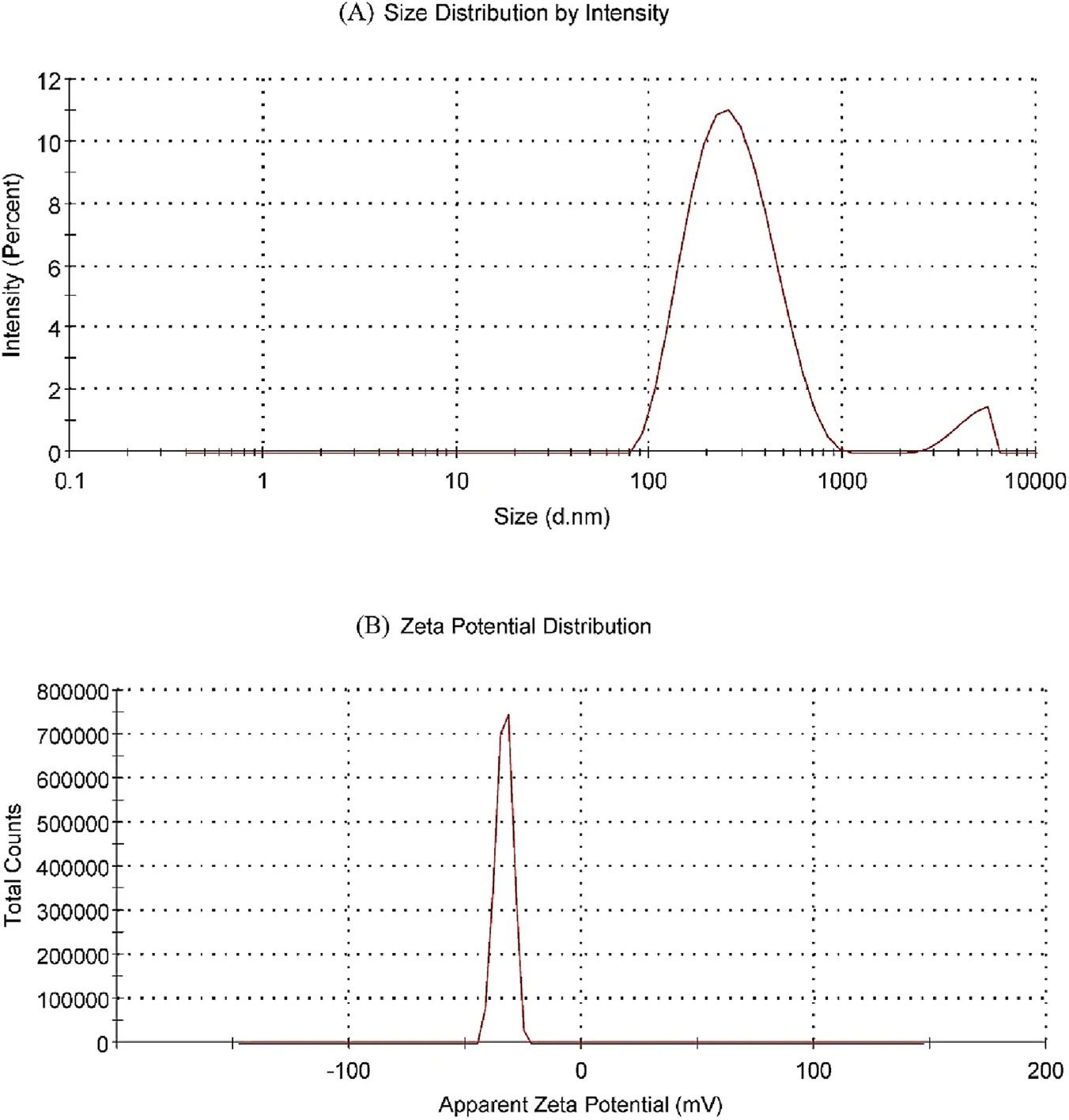
Characterization of ulvan-SeNPs. **(A)** Intensity of size distribution by dynamic light scattering (DLS), and **(B)** Zeta potential distribution.

The morphology and size distribution of the produced nanoparticles were examined using transmission electron microscopy (TEM), as shown in Figures 3A–C display representative micrographs. A uniform population of quasi-spherical nanoparticles was detected in all samples. The particles seemed dense and exhibited negligible aggregation, facilitating precise determination of individual particle sizes.

**FIGURE 3:**
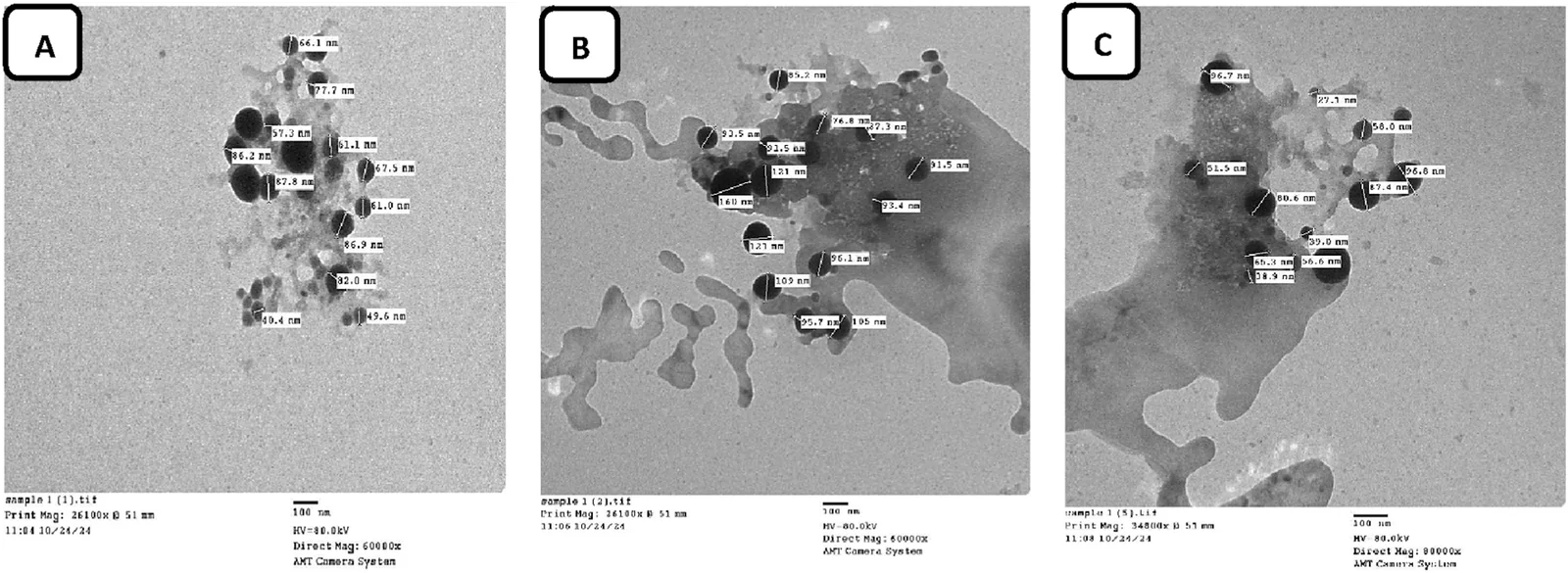
Transmission electron microscopy (TEM) images of ulvan‐mediated selenium nanoparticles (ulvan‐SeNPs). **(A–C)** represent TEM images showing the morphology and size distribution of the nanoparticles. Scale bar: 100 nm.

Particle size analysis was conducted by measuring the diameters of individual nanoparticles (n > 10 per image). Figure 3A illustrates that particles within this field of view displayed a size range of 40.4–87.8 nm, with a mean diameter of 69.5 ± 16.6 nm (mean ± standard deviation)—Figure 3B demonstrated a population characterized by a more restricted distribution, spanning from 76.8 to 160 nm, with a mean diameter of 100.1 ± 18.1 nm—the particles depicted in Figure 3C were rather diminutive, exhibiting widths 27.1: 96.8 nm, with a mean of 62.7 ± 22.7 nm. The disparity in average size among the imaged fields indicates a moderate level of polydispersity within the synthesized batch. The TEM investigation verifies the practical synthesis of nanoscale, spherical particles with sizes predominantly ranging from roughly 40 nm–120 nm.

### 3.2 Molecular docking

The affinity distribution of docking shown in Table 3 demonstrates a clear dissimilarity in the binding behavior of the ulvan-derived saccharide fragment among the evaluated protein targets. Notably, VEGFR2 (Figure 4) revealed the strongest predicted binding, as reflected by the most favorable binding energy values, followed by CD44 (Figure 5) and TLR2 (Figure 6). In contrast, LGALS3 (Figure 7) established comparatively weaker binding affinity and was therefore used as a computational reference for relative selectivity analysis.

**TABLE 3:** Best docking pose, predicted Ki, and relative selectivity index (RSI) of ulvan fragment.

Protein	Best binding energy (kcal/mol)	Predicted ki (µM)	Relative selectivity index (RSI)
CD44	−7.7	2.25	158.62
FGF2	−5.8	55.72	6.41
LGALS3	−4.7	357.10	1.00 (reference)
TLR2	−7.3	4.42	80.72
TLR4	−6.5	17.08	20.90
VEGF-A	−6.9	8.69	41.08
VEGFR2	−8.4	0.69	517.32

**FIGURE 4:**
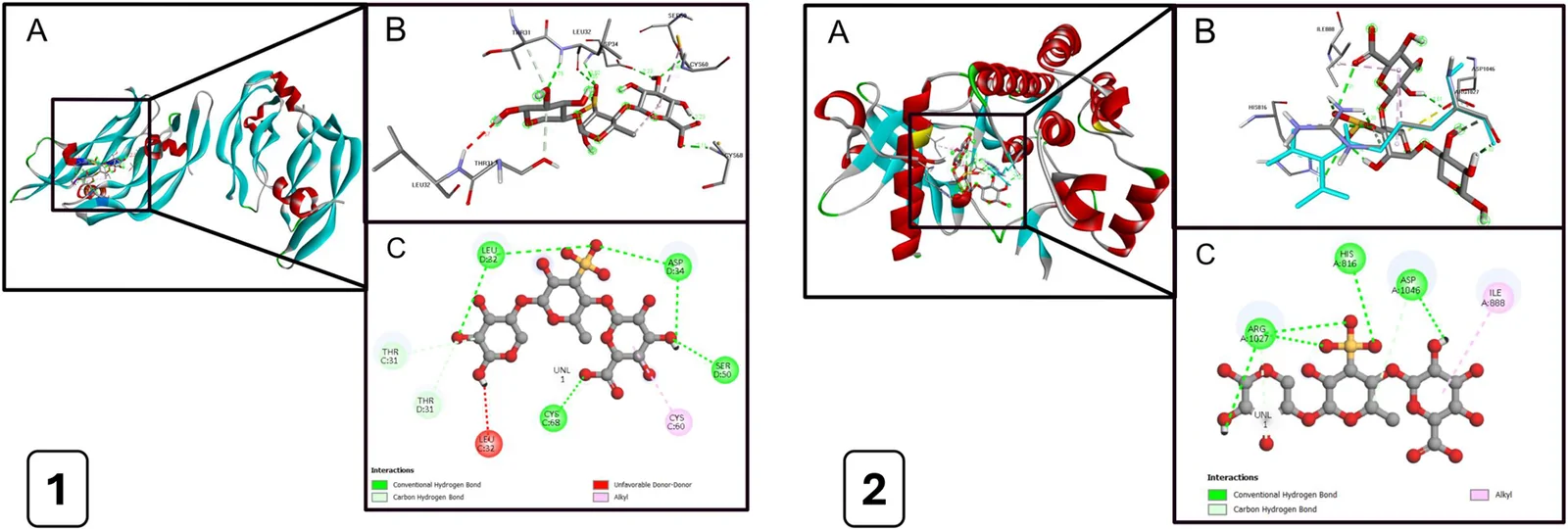
2D and 3D representation of the docked structures of the Ulvan with Angiogenesis-Related Pathways. (1) 2D and 3D interaction structure of Ulvan with VEGF-A protein. (2) 2D and 3D interaction structure of Ulvan with VEGFR2 protein.

**FIGURE 5:**
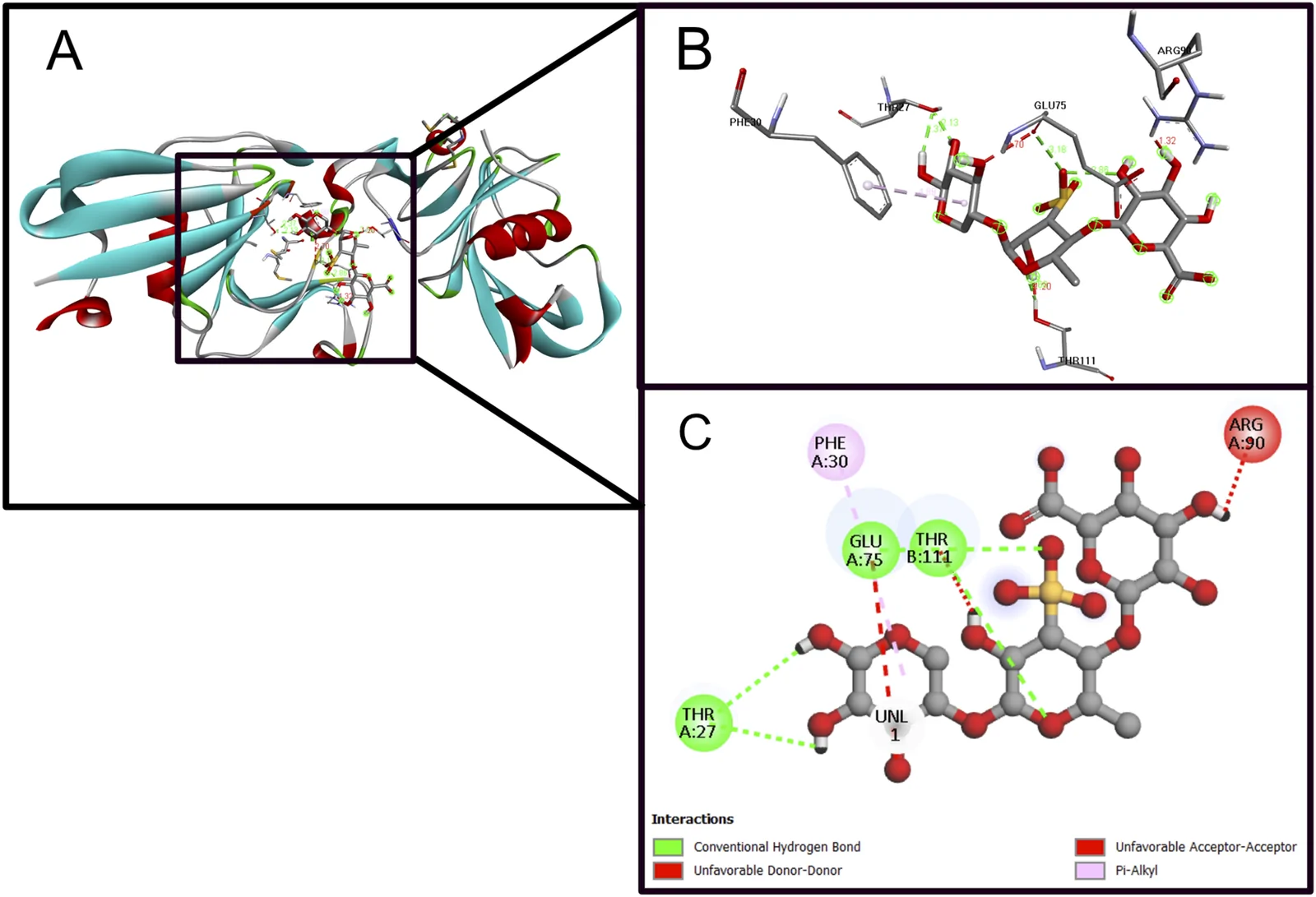
2D and 3D representation of the docked structures of the Ulvan with CD44 via Hydrogen-Bonding Interactions.

**FIGURE 6:**
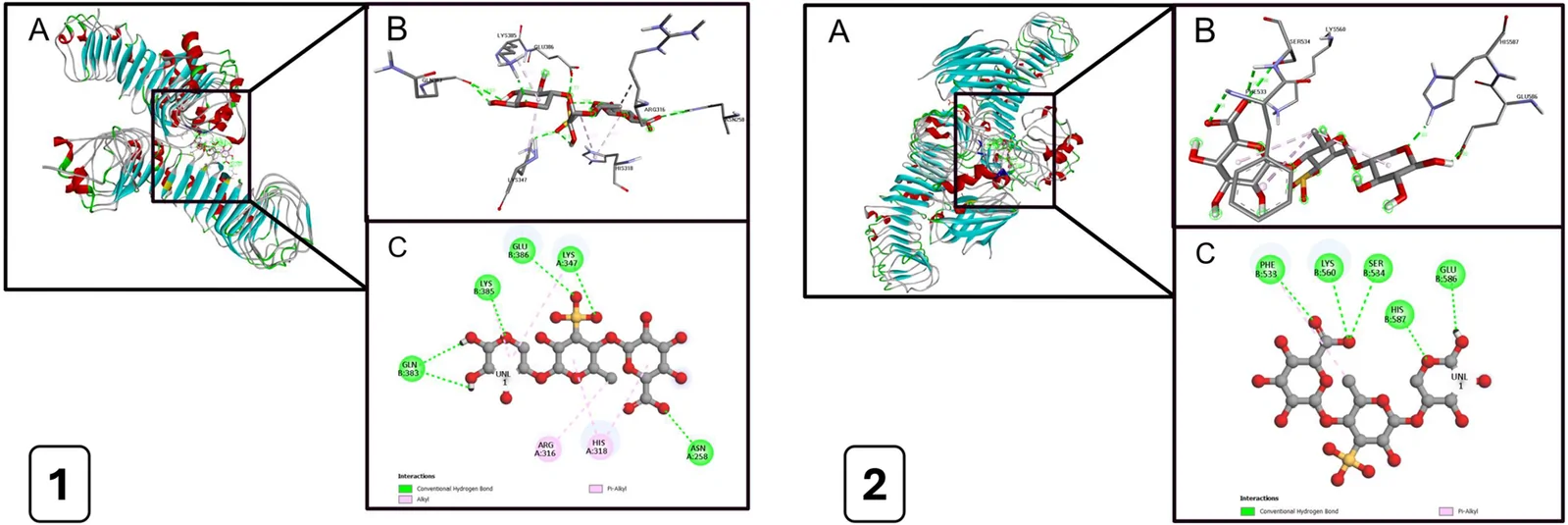
2D and 3D representation of the docked structures of the Ulvan with Innate Immune Receptors. (1) 2D and 3D interaction structure of Ulvan with TLR2 protein. (2) 2D and 3D interaction structure of Ulvan with TLR4 protein.

**FIGURE 7:**
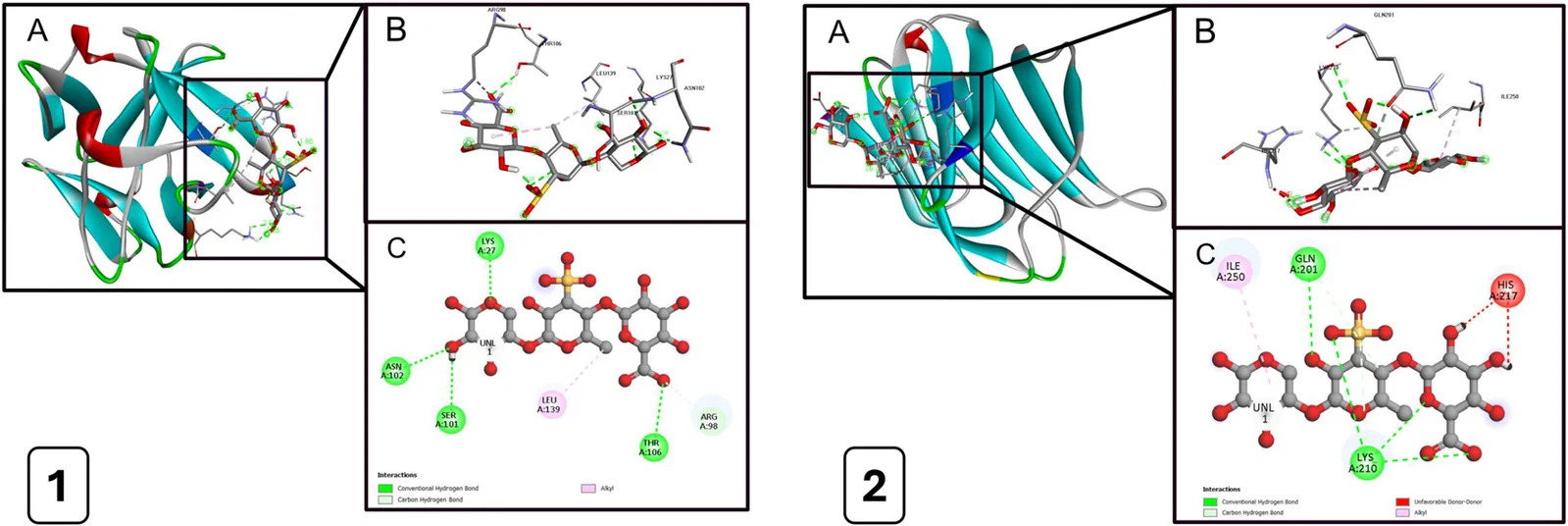
2D and 3D representation of the docked structures of the Ulvan with angiogenesis-modulating proteins. (1). 2D and 3D interaction structure of Ulvan with FGF2 protein. (2) 2D and 3D interaction structure of Ulvan with LGALS3 protein.

The calculated inhibition constants (Ki) and relative selectivity index (RSI) values further support these observations, indicating a superior interaction of the ulvan fragment with angiogenesis-related and immune-associated targets, as mentioned in Table 3. Overall, these results suggest that ulvan may exert multi-target activity through differential binding across distinct biological pathways rather than through a single dominant molecular mechanism, as shown in Figure 8.

**FIGURE 8:**
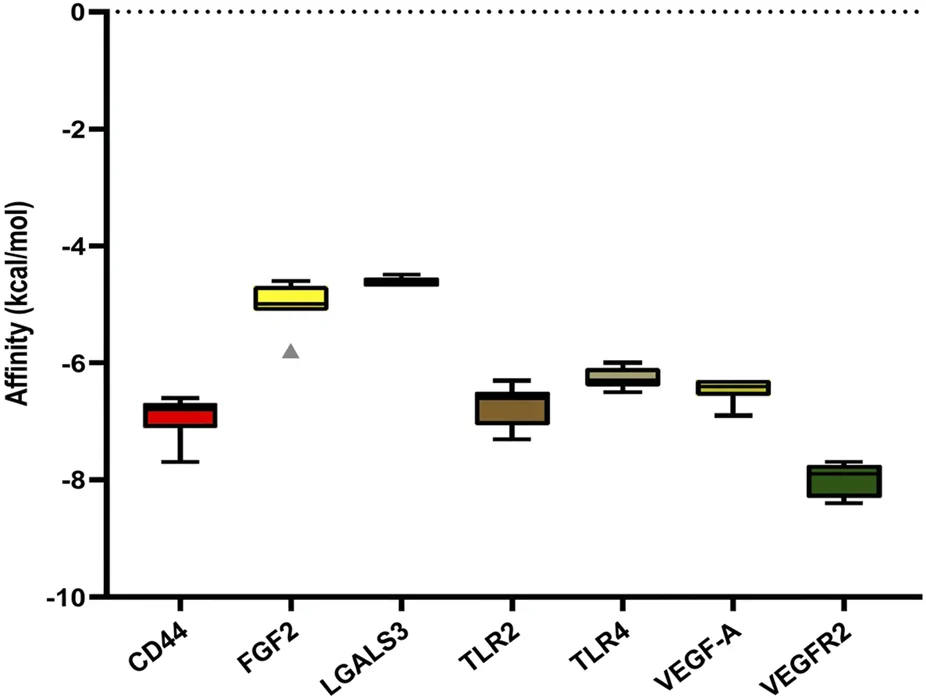
Docking affinity (kcal/mol) distribution of ulvan-derived saccharide fragments against selected targets associated with the Ehrlich tumor model. Lower affinity values correspond to stronger predicted binding.

### 3.3 Assessment of NaSe, ulvan, NanoSe, and CP effect on body weight, tumor weight, tumor volume, and tumor growth inhibition index

Tumour size was then assessed through the measurement of length and width among the groups, Control, NaSe, Ulvan, Ulvan–NanoSe, and CP, as seen in Figure 9.

**FIGURE 9:**
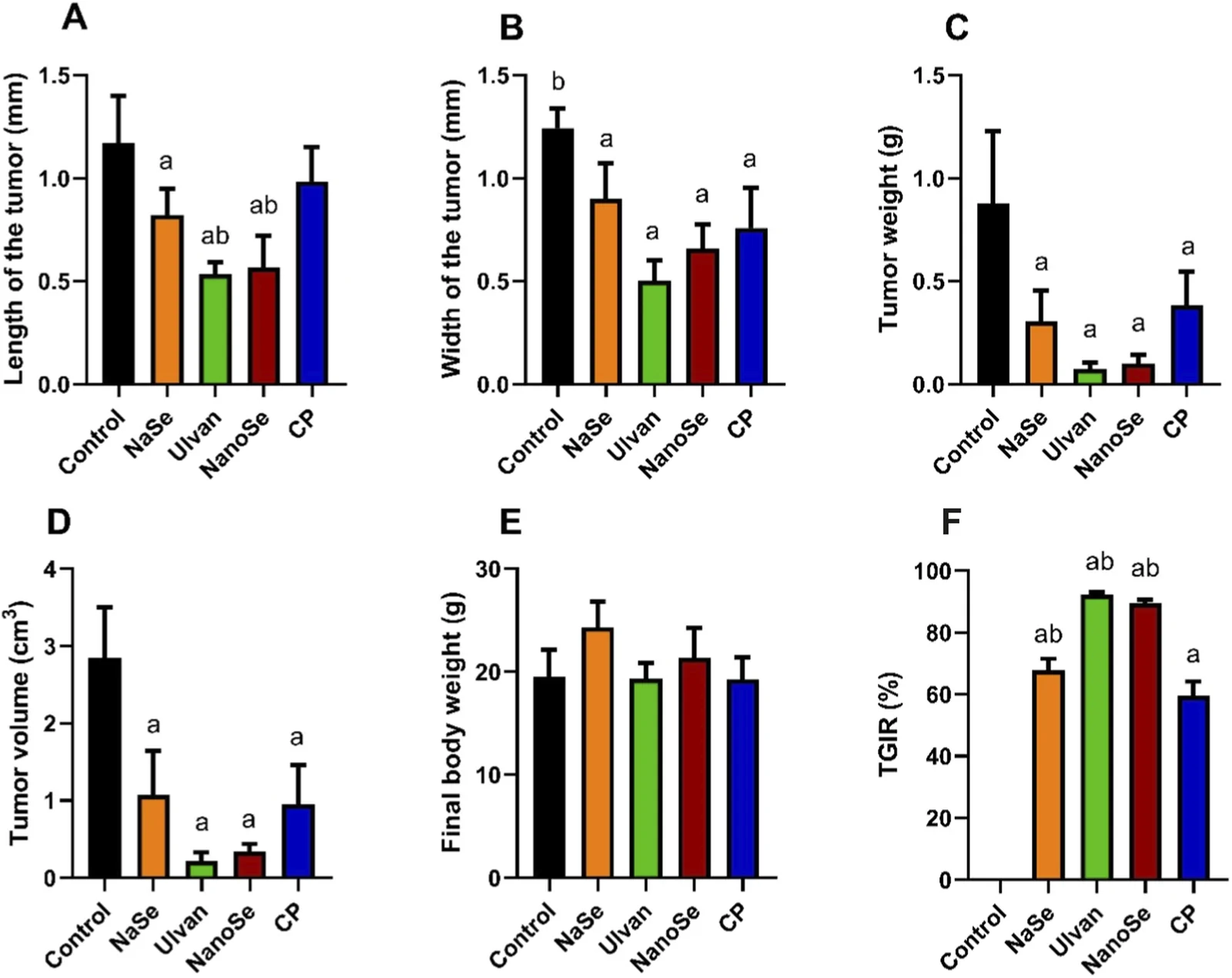
Effect of Ulvan and Ulvan–NanoSe treatments on tumor growth dimensions in the experimental groups. **(A)** Tumor length (mm). **(B)** Tumor width (mm). **(C)** Tumor weight. **(D)** Tumor volume. **(E)** Final body weight. **(F)** Tumor growth inhibition rate (TGIR). (a) p < 0.05 vs. control mice; (b) p < 0.05 vs. CP mice. These annotations indicate statistically significant differences between groups.

As shown in Figure 9, treatment with NaSe, Ulvan, NanoSe, and CP resulted in significant (p < 0.05) reductions in tumour growth compared with the Control group. The reduced tumor growth was observed in tumor length (A), tumor width (B), tumor weight (C), and tumor volume (D).

Among the treated groups, Ulvan and NanoSe showed the highest reduction in tumor length, tumor width, and tumor volume. The tumor weight was also reduced significantly in all treated groups compared with the Control group. The post-hoc pairwise comparison of Duncan’s test showed that the difference in tumor weight between the NanoSe and CP groups was statistically non-significant (*P* = 0.4048), which indicates that the nano-selenium formulation possesses comparable antitumor efficacy with the standard chemotherapeutic agent. Similarly, the tumor volume comparison of NanoSe and CP did not show significant difference (*P* = 0.4511). All other pairwise comparisons were significantly different (*P* < 0.05).

The final body weight (E) did not show significant adverse effects in treated groups compared with the Control group. The tumor growth inhibition rate (TGIR%) (F) also confirmed the above results. Ulvan and NanoSe showed the highest inhibition rates compared with other treated groups. The inhibition rates were followed by NaSe and then CP. Ulvan and NanoSe showed the highest antitumor effects compared with other treated groups.

### 3.4 Oxidative stress and antioxidant biomarkers in the ESC muscle tissue

#### 3.4.1 Oxidative stress markers

The control group demonstrated markedly increased levels of lipid peroxidation (LPO) and nitric oxide (NO), indicating substantial oxidative stress linked to tumor advancement. Treatment with sodium selenite led to a modest decrease in LPO levels alongside an increase in NO generation (*p*-value = 0.994 and 0.0011, respectively). On the other hand, ulvan therapy significantly diminished both LPO and NO levels compared to the control group (*p*-values = 0.0000548 and 0.00031, respectively). Nano-selenium therapy occasioned a significantly minor decrease in LPO and a non-significant decrease in NO oxidative stress indicators (*p*-value = 0.0234 and 0.428, respectively), whereas cisplatin treatment markedly elevated LPO and NO levels, indicating greater oxidative damage (*p*-value = 0.00000281 and 0.000000113, respectively) (Figure 10).

**FIGURE 10:**
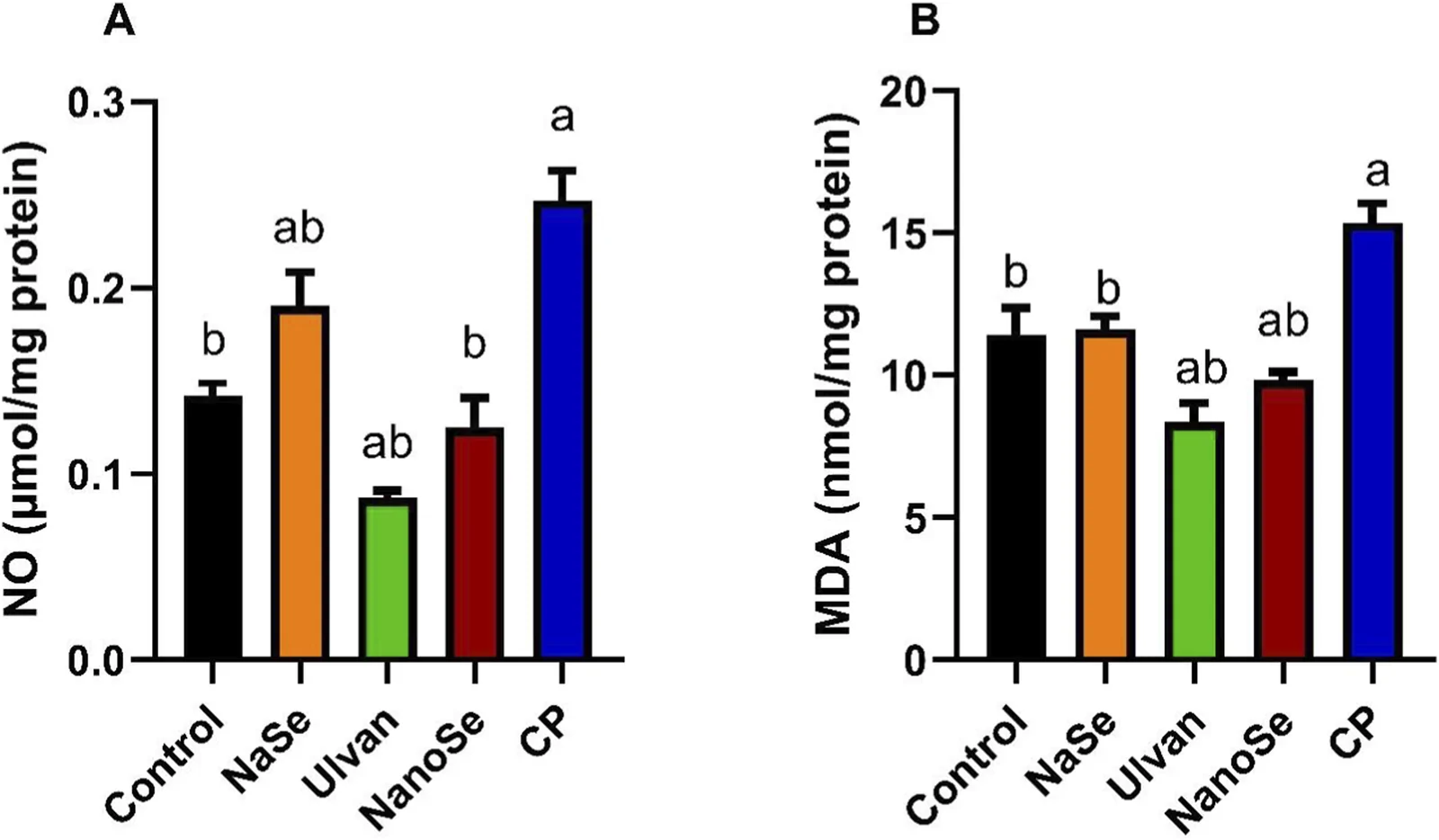
Effects of NaSe, Ulvan, and NanoSe treatments on NO and lipid peroxidation levels. Biochemical data are expressed as mean ± SEM (n = 7). Distinct superscript letters indicate statistically significant differences between groups (p < 0.05). **(A)** p < 0.05 vs. control mice; **(B)** p < 0.05 vs. CP mice. These annotations indicate statistically significant differences between groups.

#### 3.4.2 Antioxidant enzyme activities

The activities of superoxide dismutase (SOD), catalase (CAT), glutathione peroxidase (GPx), glutathione reductase (GRd), and the quantity of reduced glutathione (GSH) were markedly diminished in the control group relative to normal physiological values. The administration of sodium selenite resulted in a moderate enhancement of antioxidant enzyme activity (*p*-value = 0.0759, 0.0012, 0.0013, 0.0003, and 0.9011, respectively). Ulvan treatment significantly restored antioxidant defenses (*p*-value = 0.8970, 0.1832, 0.7383, 0.1040, and 0.9929, respectively), but nano-selenium treatment produced the most significant boost of antioxidant enzyme activity among all groups treated (p-value = 0.0003, >0.9999, <0.0001, <0.0001, and 0.4222, respectively). Cisplatin therapy decreased antioxidant enzyme activity, indicating disrupted redox homeostasis (*p*-value = 0.1716, 0.0664, 0.2071, 0.3354, and 0.0021, respectively) (Figure 11). Total protein in the ESC tissue was significantly increased by treatment with nano-selenium when compared to the control and cisplatin groups (*p*-value = 0.001and 0.0002, respectively), while the administration of sodium selenite showed a moderate increase in comparison to the cisplatin group (*p*-value = 0.0259) (Figure 12).

**FIGURE 11:**
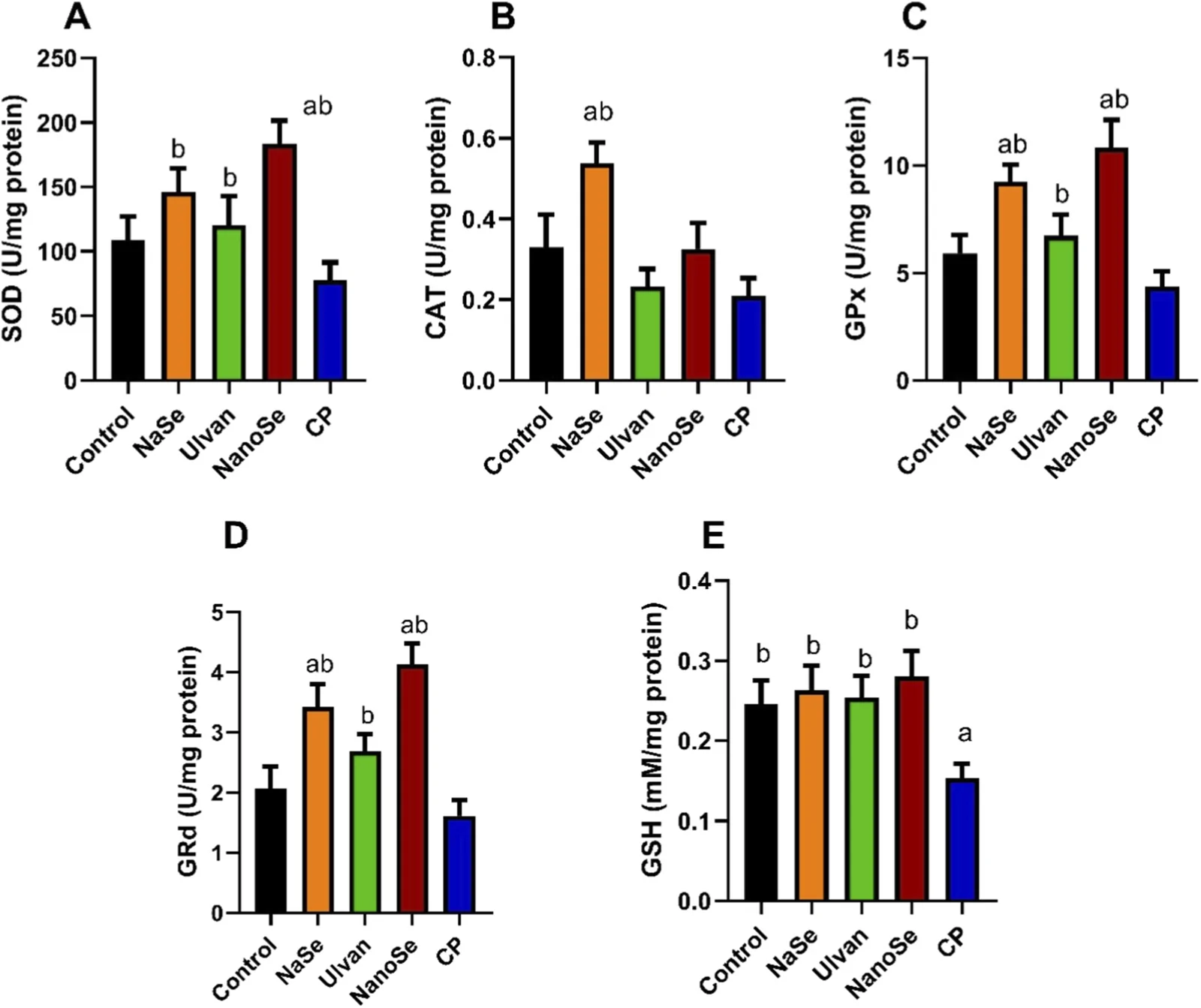
The impact of cisplatin (CP), Ulvan, NanoSe, and NaSe on biomarkers linked to redox and antioxidant processes. Glutathione peroxidase (GPx), glutathione reductase (GRd), reduced glutathione (GSH), catalase (CAT), and superoxide dismutase (SOD) activities or levels are shown in panels **(A–E)**. Biochemical data are expressed as mean ± SEM (n = 7). Distinct superscript letters indicate statistically significant group differences (p < 0.05). (a) p < 0.05 vs. control mice; (b) p < 0.05 vs. CP mice reflect the statistically significant differences between groups.

**FIGURE 12:**
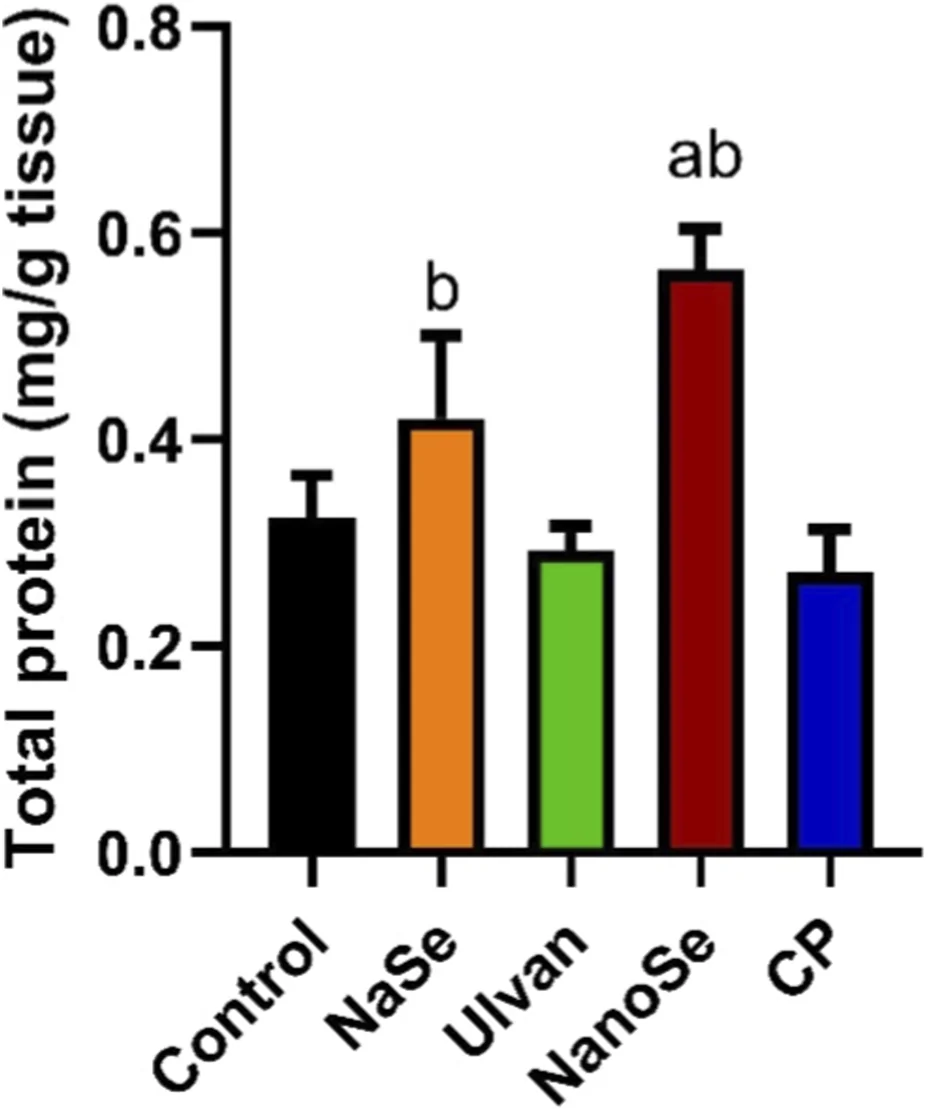
The impact of cisplatin (CP), Ulvan, NanoSe, and NaSe on total protein content in the ESC tissue. Biochemical data are expressed as mean ± SEM (*n* = 7). Distinct superscript letters indicate statistically significant group differences (*p* < 0.05). (a) *p* < 0.05 vs. control mice; (b) *p* < 0.05 vs. CP mice reflect the statistically significant differences between groups.

### 3.5 Inflammatory mediators

Levels of tumor necrosis factor-α (TNF-α), nuclear factor kappa B (NF-κB), and interleukin-1β (IL-1β) were markedly normal in the control group, as illustrated in Figure 13. The administration of sodium selenite resulted in a moderate elevation in these inflammatory mediators (*p*-value = 0.1107, 0.0668, and 0.0076, respectively). Ulvan treatments reduced levels of TNF-α, NF-κB, and IL-1β in comparison to the control group (*p*-value = 0.9998, 0.9158, and 0.7955, respectively), while nano-selenium treatments significantly elevated levels of TNF-α, NF-κB, and IL-1β in comparison to the control group (p-value = 0.0181, 0.0012, and 0.0002, respectively). In contrast, cisplatin therapy significantly increases inflammatory responses and sustained high levels of inflammatory markers (*p*-value = 0.0003, <0.0001, and <0.0001, respectively).

**FIGURE 13:**
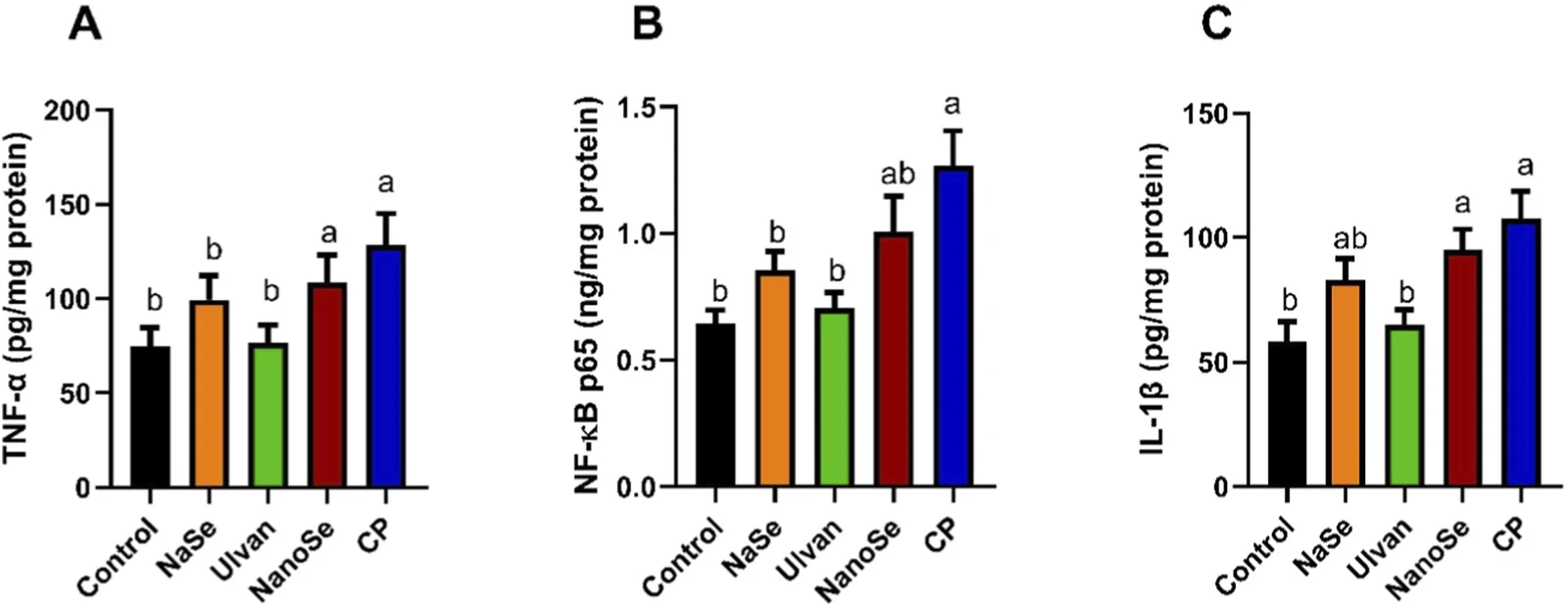
The impact of cisplatin (CP), Ulvan, NanoSe, and NaSe on important inflammatory mediators. Tumor necrosis factor-α (TNF-α), nuclear factor-κB p65 (NF-κB p65), and interleukin-1β (IL-1β) tissue levels are displayed in panels **(A–C)**. Biochemical data are expressed as mean ± SEM (*n* = 7). Distinct superscript letters indicate statistically significant group differences (*p* < 0.05). (a) *p* < 0.05 vs. control mice; (b) *p* < 0.05 vs. CP mice reflect the statistically significant differences between groups.

### 3.6 Apoptotic biomarkers

The control group demonstrated a notable elevation in the anti-apoptotic protein Bcl-2 and a reduction in pro-apoptotic Bax levels, leading to a diminished Bax/Bcl-2 ratio, as shown in Figure 14. Treatment with sodium selenite resulted in a significant elevation of Bax levels and a significant reduction in Bcl-2 expression (*P-value* 0.0109 and 0.0297, respectively). While ulvan resulted in a moderate, non-significant elevation of Bax levels and a decrease in Bcl-2 expression (*P*-value = 0.2893 and 0.3255, respectively). Nano-selenium treatment considerably boosted Bax levels, decreased Bcl-2 expression, and substantially elevated the Bax/Bcl-2 ratio (*P*-value = 0.0010, 0.0135, and <0.0001, respectively). The activity of caspase-3 was significantly elevated in the groups treated with nano-selenium and cisplatin (*P*-value= <0.0001 and <0.0001, respectively), signifying a strong activation of apoptotic pathways.

**FIGURE 14:**
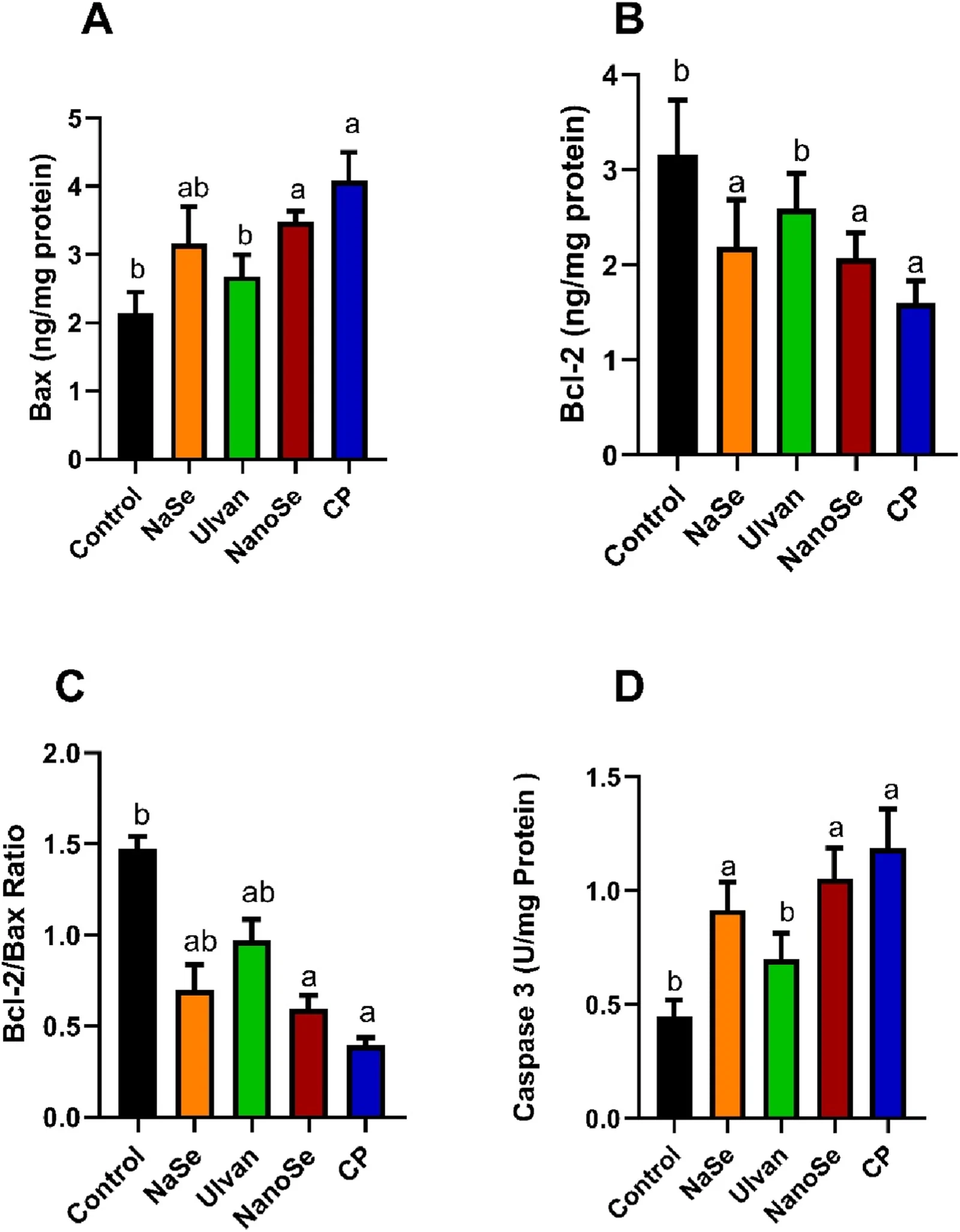
The impact of cisplatin (CP), Ulvan, NanoSe, and NaSe on important apoptotic markers. Bax, Bcl-2, Bcl-2/Bax ratio, and caspase-3 tissue levels are displayed in panels **(A–D)**. Biochemical data are expressed as mean ± SEM (*n* = 7). Distinct superscript letters indicate statistically significant group differences (*p* < 0.05). (a) *p* < 0.05 vs. control mice; (b) *p* < 0.05 vs. CP mice reflect the statistically significant differences between groups.

### 3.7 Autophagy-related gene expression

The relative mRNA expression levels of autophagy-related markers, namely Beclin-1, mTOR, ATG5, and ATG7, were evaluated across all experimental groups (Control, NaSe, Ulvan, NanoSe, and CP) (Figures 15A–D). Beclin-1 expression levels were upregulated in all treated groups compared with the control group (Figure 15A). The NanoSe group showed maximum upregulation, followed by NaSe and Ulvan, whereas CP showed moderate upregulation. Conversely, mTOR expression levels were downregulated in all treated groups compared to the control group (Figure 15B). The NanoSe group showed the greatest downregulation (*P* value = 0.0093), suggesting possible activation of autophagy, followed by NaSe, Ulvan, and CP, which showed moderate downregulation. For ATG5 expression, all treated groups showed upregulation of the gene compared to the control group (Figure 15C). The NanoSe group showed maximum upregulation (*P* value = 0.0171), followed by NaSe, Ulvan, and CP, showing moderate upregulation. Similar results were observed for ATG7 expression, where all treated groups showed upregulation of the gene compared to the control group (Figure 15D). The NanoSe group showed maximum upregulation (*P* value = 0.0261), showing statistical significance compared to all other groups, whereas NaSe, Ulvan, and CP showed moderate upregulation.

**FIGURE 15:**
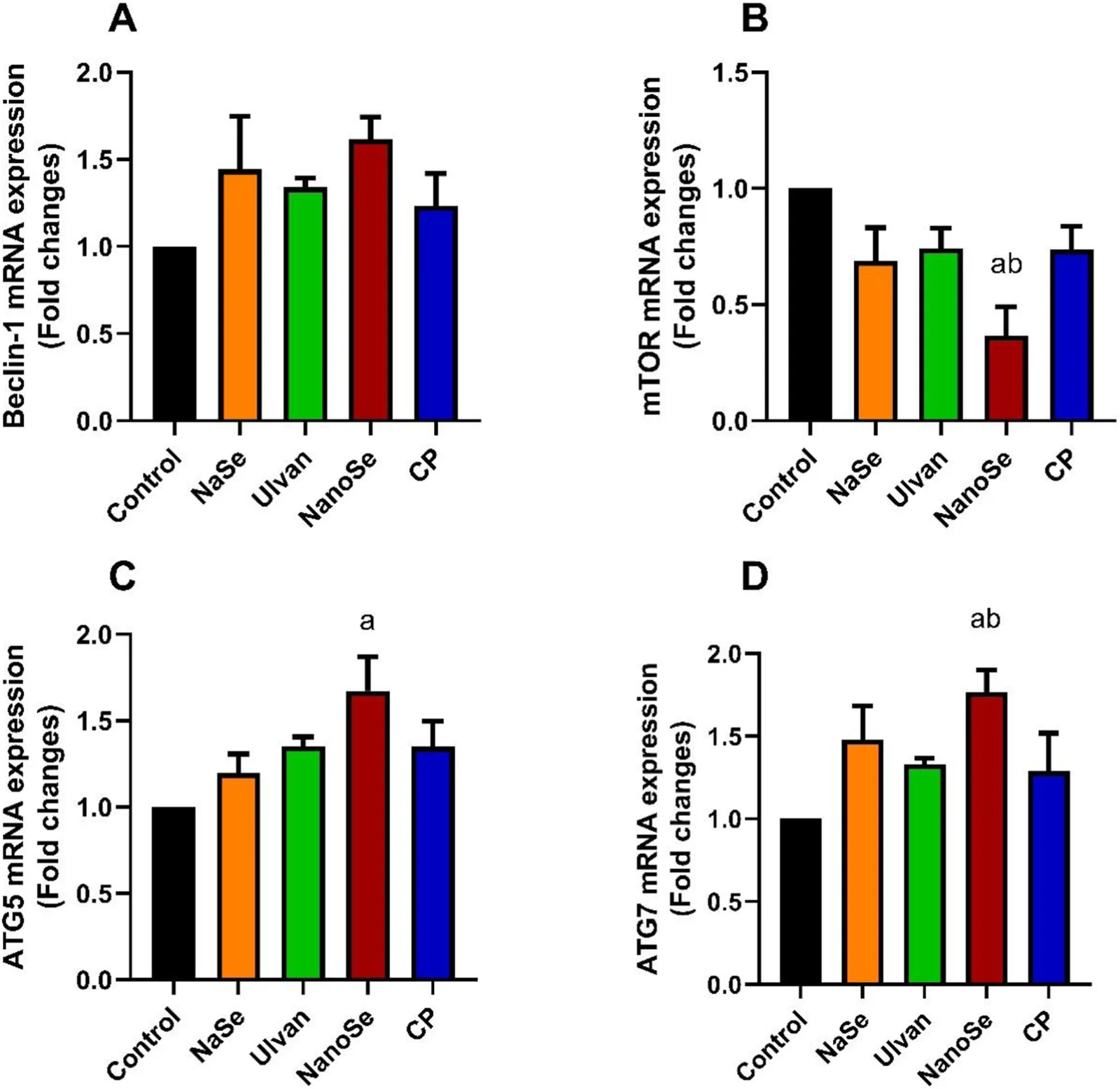
Effects of NaSe, Ulvan, NanoSe, and cisplatin (CP) on the mRNA expression levels of autophagy‐related genes in Ehrlich solid carcinoma (ESC) tissue. **(A)** Beclin‐1, **(B)** mTOR, **(C)** ATG5, and **(D)** ATG7. Data are presented as mean ± SEM. Different superscript letters indicate statistically significant differences between groups (p < 0.05).

### 3.8 Histopathological and immunohistochemical examinations

#### 3.8.1 Histopathological examination

The histopathological examination of Ehrlich solid carcinoma (ESC) muscle sections stained with hematoxylin and eosin (H&E) showed variation among different groups as presented in Figure 16.

**FIGURE 16:**
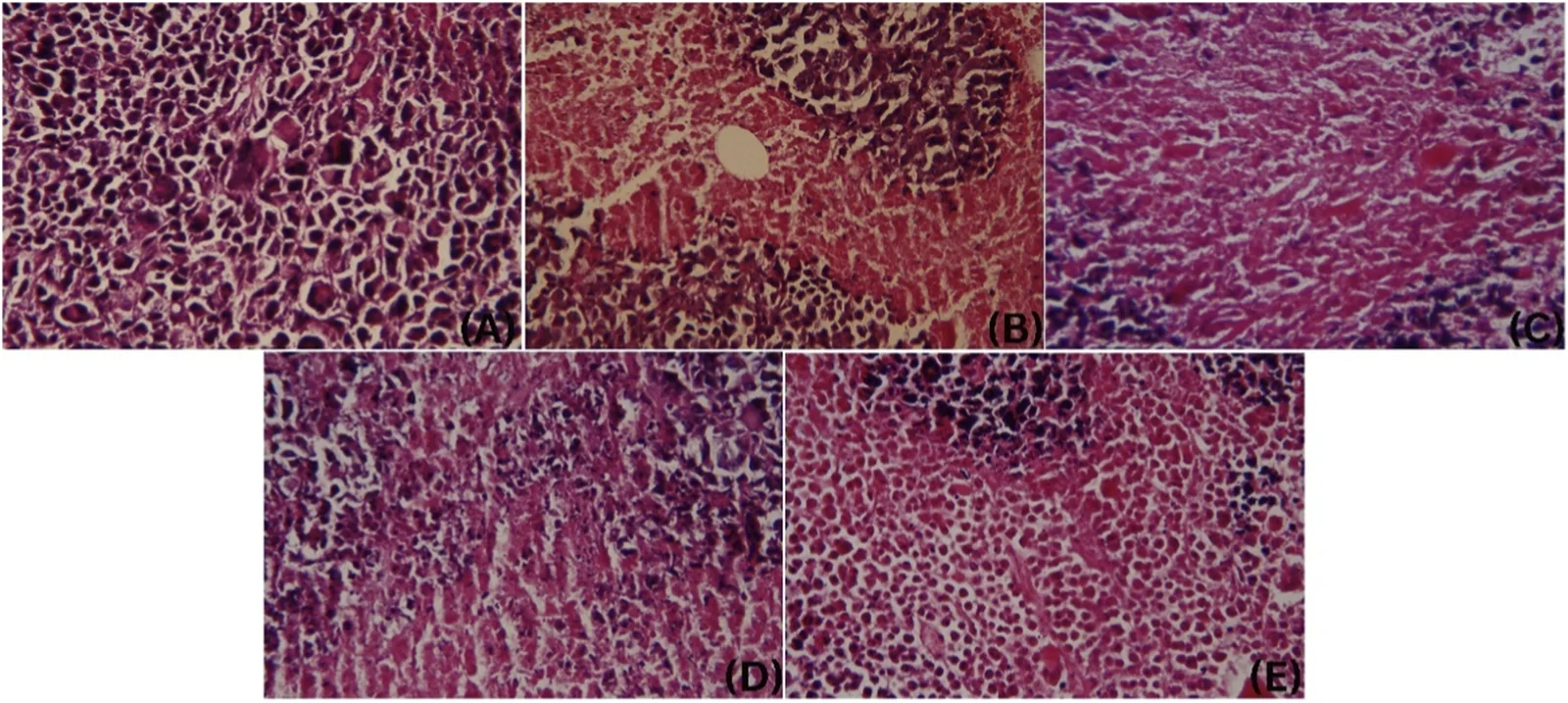
Histopathological analysis of Ehrlich solid carcinoma (ESC) muscle tissue slices stained with hematoxylin and eosin (H&E). **(A)** control group, **(B)** sodium selenite-treated group (NaSe), **(C)** ulvan-treated group, **(D)** nano-selenium-treated group, and **(E)** cisplatin-treated group (CP).

In the control group (A), the ESC sections showed intense infiltration of densely packed malignant cells, leading to extreme modification of the normal architecture of the muscles. The tumor cells showed extreme pleomorphism, hyperchromasia, an enhanced nuclear to cytoplasmic ratio, and numerous mitoses, indicating intense proliferation. In the sodium selenite-treated group (NaSe) (B), there was a moderate decrease in the number of tumor cells compared to the control. The tumor cells showed mild degenerative changes, as indicated by cytoplasmic vacuolations and patchy nuclear condensations. Some preservation of muscle fibers with moderate infiltration of inflammatory cells indicated limited anti-tumor effects. Samples from the ulvan-treated group (C) showed a considerable reduction in the infiltration of tumors, in addition to a partial recovery of the muscle tissue architecture. Tumor cells showed degenerative changes, such as nuclear pyknosis and eosinophilia, together with scattered necrotic areas. However, there was significantly reduced infiltration of the inflammatory cells, showing both potent anticancer and anti-inflammatory effects. Histopathological studies in the nano-selenium-treated group (D) showed considerable reduction in the mass of the tumor, in addition to its viable cells. There was substantial cellular degeneration and necrosis, together with the retention of muscle tissue architecture. However, there was evidence of distinct apoptotic changes in the tumor cells, characterized by nuclear fragmentation and the shrinking of cells, showing substantial anticancer potency with minimal tissue damage. The cisplatin-treated group (CP) (E) showed considerable necrosis in the tumor, in addition to its viable cell mass. However, this potent anticancer action was accompanied by substantial tissue injury, such as the loss of muscle tissue architecture, bleeding, and infiltration of the inflammatory cells, showing the cytotoxic aspects of cisplatin therapy.

#### 3.8.2 Immunohistochemistry examination

Immunohistochemical examination demonstrated significant cytoplasmic VEGF expression (Figure 17) and pronounced nuclear and cytoplasmic NF-κB (Figure 18) positivity in the control group, signifying increased angiogenesis and inflammatory signaling. Treatment with sodium selenite and ulvan led to moderate decreases in VEGF and NF-κB expression. Nano-selenium therapy significantly inhibited VEGF and NF-κB immunoreactivity, while cisplatin exhibited diminished expression linked to considerable tissue damage. LC3 immunostaining (Figure 19) was negligible in the control group, whereas sodium selenite and ulvan treatments elicited substantial LC3 expression. Conversely, nano-selenium therapy demonstrated pronounced LC3 immunopositivity, indicating increased autophagy-related activity. Variable expression of LC3 was noted in the cisplatin group.

**FIGURE 17:**
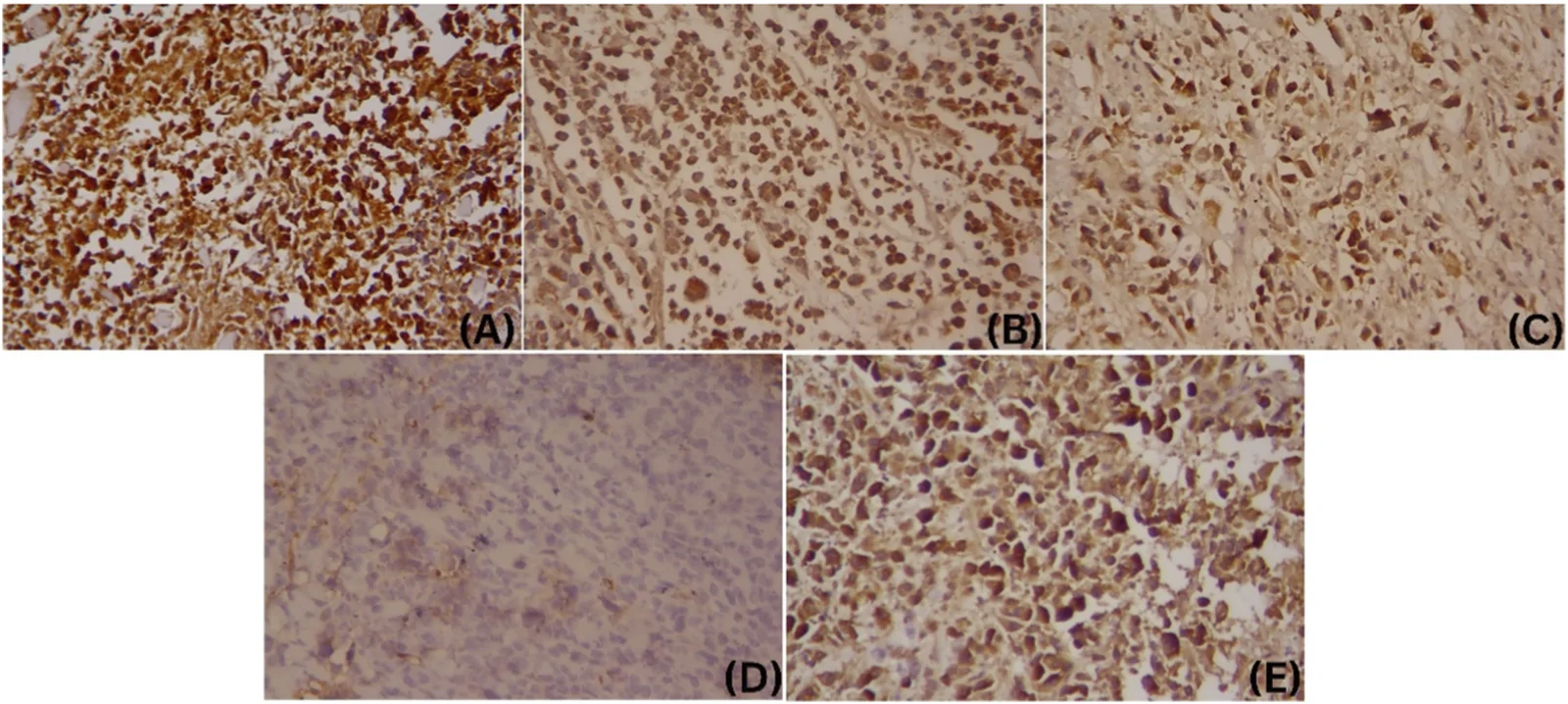
Immunohistochemical analysis of Ehrlich solid carcinoma (ESC) muscle tissue slices stained with cytoplasmic VEGF. **(A)** control group, **(B)** sodium selenite-treated group (NaSe), **(C)** ulvan-treated group, **(D)** nano-selenium-treated group, and **(E)** cisplatin-treated group (CP).

**FIGURE 18:**
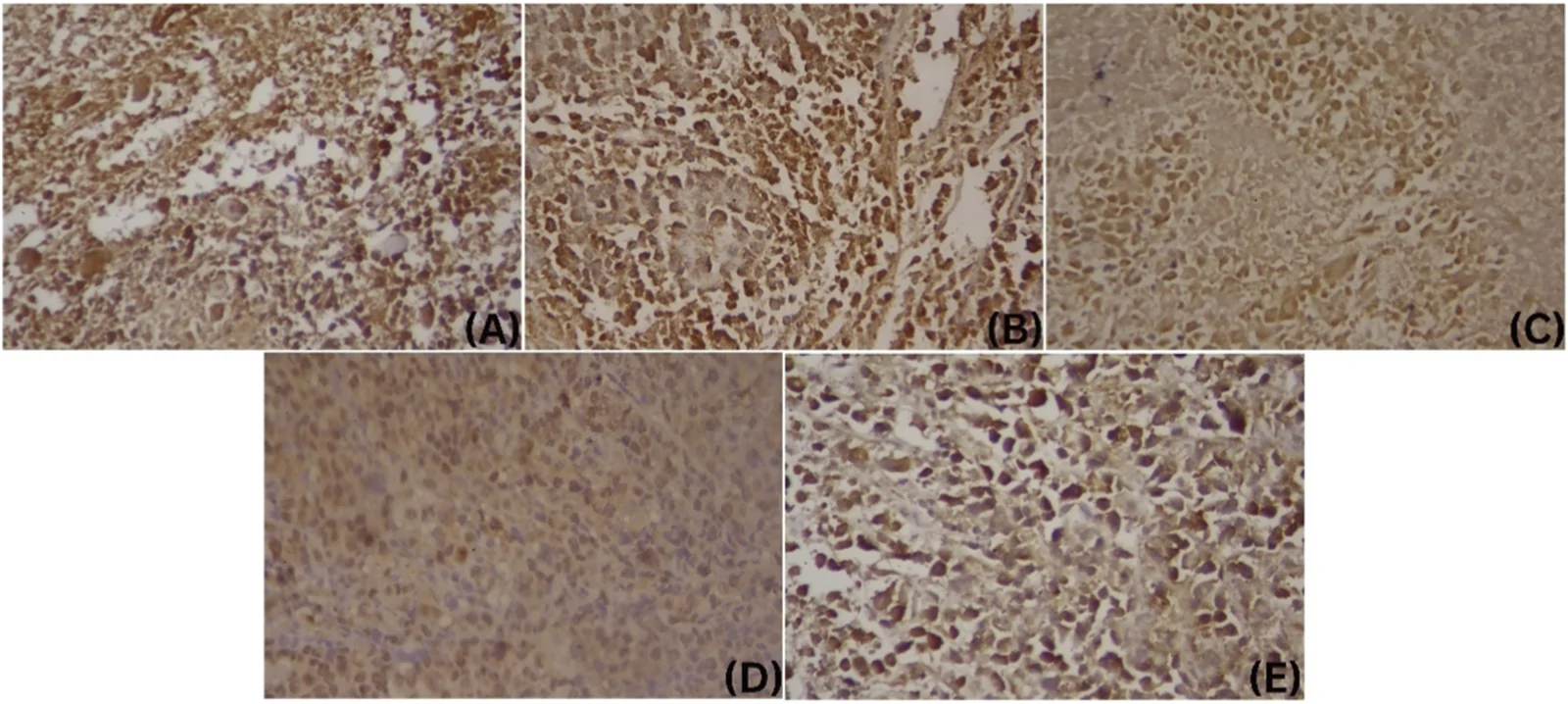
Immunohistochemical analysis of Ehrlich solid carcinoma (ESC) muscle tissue slices stained with pronounced nuclear and cytoplasmic NF-κB. **(A)** control group, **(B)** sodium selenite-treated group (NaSe), **(C)** ulvan-treated group, **(D)** nano-selenium-treated group, and **(E)** cisplatin-treated group (CP).

**FIGURE 19:**
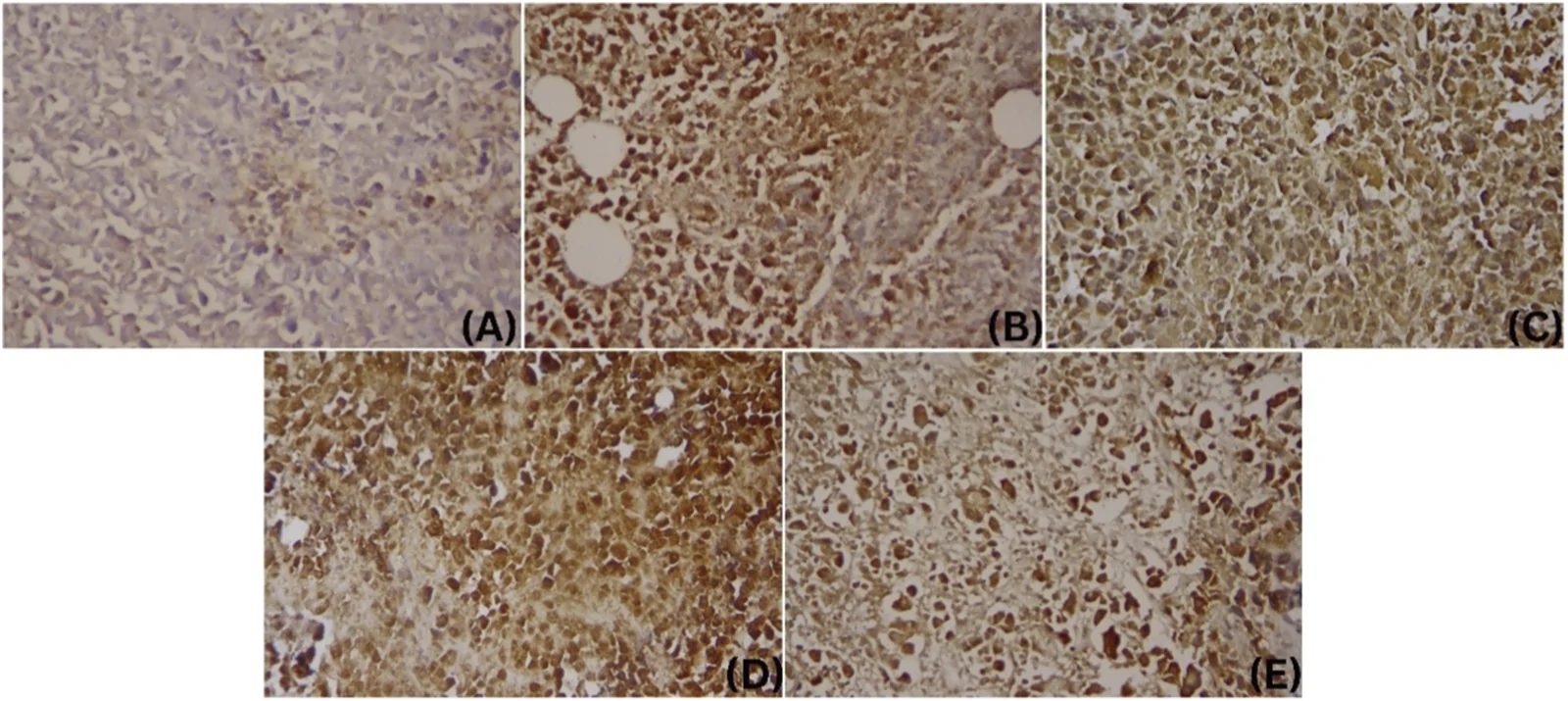
Immunohistochemical analysis of Ehrlich solid carcinoma (ESC) muscle tissue slices stained with LC3 immunostaining. **(A)** control group, **(B)** sodium selenite-treated group (NaSe), **(C)** ulvan-treated group, **(D)** nano-selenium-treated group, and **(E)** cisplatin-treated group (CP).

## 4 Discussion

This paper presents a systematically integrated, mechanistically grounded assessment of the anti-tumor efficiency of ulvan and nanoselenium carrying ulvan in the ESC model. Unlike previous studies, which may involve isolated findings in biochemistry, molecular biology, and histopathology, our results establish a causally continuous sequence from *in silico* target engagement to the reprogramming of cellular signaling pathways, redox balance, inflammation, the initiation of programmed cell death, and ultimately, structural regression of the tumor. This multi-level consistency strongly suggests that the noted anti-tumor effects are, in fact, not random events, but a coordinated biological response prompted by ulvan and further potentiated by its nanoselenium preparation.

Molecular docking studies indicate a Preferential binding of ulvan-derived saccharides to several tumor-related targets, especially VEGFR2, CD44, and Toll-like receptors TLR2 and TLR4. This provides a molecular rationale for direct anti-angiogenic action, as VEGFR2 is the primary signaling component that mediates endothelial cell proliferation, permeability, and neovascularization in solid tumors in response to VEGF. This prediction was corroborated *in vivo* by the marked reduction in VEGF immunoreactivity observed in ulvan- and nano-selenium–treated tissues. As angiogenesis is a host requirement for continued tumor growth, nutritional supply, and metastatic dissemination, it is anticipated that inhibition of VEGFR2 signaling inhibits tumor expansion early rather than mere secondary vascular collapse.

Thus, the resultant histological pattern of architectural remodeling in tumors treated with ulvan can be explained by the hypothetical binding of ulvan to CD44. CD44 is believed to be a critical modulator of tumor cell adhesion, anchorage of the extrymatrix, migration, and survival signaling (Cirillo, 2023; Szatanek and Baj-Krzyworzeka, 2021). Its inhibition has been associated with reduced tumor invasiveness, impaired stromal interaction, and heightened sensitivity to apoptotic stimuli (Cirillo, 2023; Senbanjo and Chellaiah, 2017). The marked reduction of tumor cell density, accompanied by partial restoration of the original organization of muscle fibers in tissues exposed to ulvan, would therefore suggest that this polysaccharide interferes with anchorage-dependent survival mediated through CD44 and thus sensitizes tumor cells to programmed death rather than a necrotic collapse (Mollaei et al., 2021).

The predicted binding of ulvan to both TLR2 and TLR4 is a key mechanism-oriented result. These pattern recognition receptors represent the top layer of any pro-inflammatory and immune-regulatory pathway, and the activation of these receptors is the dominant initiator of NF-κB signaling. NF-κB activation sustains a pro-carcinogenic microenvironment through induction of cytokine secretion, angiogenesis, oxidative stress, immune evasion, and apoptosis resistance (Nakase and Shimomori, 2025; Ma et al., 2024). The downregulation of TNF-α, IL-1β, and NF-κB p65 in ulvan-treated tissues is therefore in agreement with the hypothesis of an inhibition at the level of TLR-mediated signaling (Wang Y. et al., 2023; Guo et al., 2024). This may indicate that the removal of pro-inflammatory mediators is only one aspect and suggests that ulvan targets the level of pattern recognition receptor activation and thus extinguishes a pro-carcinogenic transcriptional program mediated by NF-κB (Alfaro et al., 2022; Al et al., 2024).

Redox dysregulation is a key feature of malignant progression. This paradoxical oxidative phenotype is established in cancer cells as they increase their levels of reactive oxygen and nitrogen species to induce genomic instability, angiogenesis, metabolic shift, and immunosuppression. Moreover, they activate stress response pathways to protect themselves from apoptosis. In untreated mice bearing ESCs, this phenotype was apparently established because they had shown significant induction of lipid peroxide (LPO) and nitric oxide (NO) with the scarcity of antioxidant defenses. This redox dysregulation is not merely associated with malignancy; it is rather a stimulator of tumor progression as well (Bellanti et al., 2025; Ranbhise et al., 2025).

Ulvan treatment caused a marked attenuation of this oxidative phenotype. The attenuation in LPO and NO levels, along with the normalization of SOD, CAT, GPx, GRd, and GSH activity, reflects the attenuation of the redox state from that associated with survival and maintenance to a more physiological/constitutive state. From a mechanistic perspective, this would seem to relate to the attenuation of upstream signaling for the NF-κB/TLR signaling pathway, leading to the attenuation of iNOS and ROS-generating enzymes. However, normalization of the redox status would reduce an important apoptosis barrier, in which excessive ROS would activate survival signaling, and not apoptosis (Circu and Aw, 2010; Alhaj Sulaiman and Katanaev, 2025; Kuo et al., 2022).

Notably, this redox normalization was not a result of indiscriminate oxidative damage. On the contrary, cisplatin, which caused known indiscriminate DNA damage and mitochondrial dysfunction (Mariconda et al., 2025; Cecati et al., 2026), severely aggravated oxidative damage, as would be predicted from its mechanism of action that leads to regression of tumors but also causes massive damage to normal tissues (Katanić Stanković et al., 2023; Tchounwou et al., 2021), which was also confirmed by histology. The effect of ulvan, on the contrary, was a result of controlled redox modulation and not just massive cytotoxicity.

Chronic inflammation represents the second key driving force of tumor development (Rembiałkowska et al., 2025; Yuan et al., 2024). High concentrations of TNF-α, IL-1β, and NF-κB within ESCs also demonstrate the existence of an autonomous inflammatory microenvironment (Li et al., 2023; Wang et al., 2025). There was a profound reduction of these factors, as expected based on the predicted binding of ulvan to TLR2/4. Given the capability of NF-κB to transcriptionally modulate the whole spectrum of anti-apoptotic proteins, including the bcl-2 protein, its inhibition should therefore destroy the key survival pathway within cancer cells (Pari et al., 2025; Pappou et al., 2022).

The disparity between ELISA and IHC results for NF-κB in the nano-selenium-treated group requires careful interpretation. ELISA measurements reflect total tissue levels of NF-κB p65, which may include active nuclear translocated pools, inactive cytoplasmic stores, and secreted inflammatory mediators. Conversely, IHC mainly detects the activated, translocated form of NF-κB in specific cell types. The increased total NF-κB levels measured by ELISA after nano-selenium treatment likely reflect the general cellular stress response and increased protein synthesis. The decreased immunoreactivity detected by IHC specifically indicates reduced nuclear translocation and transcriptional activation of NF-κB in tumor cells. This is consistent with the known pro-oxidative mechanism of selenium, which can increase total protein expression while inhibiting nuclear translocation of transcription factors. The elevated levels of TNF-α and IL-1β detected by ELISA also favor an active immune response rather than a sustained pro-inflammatory signaling.

This mechanistic link was clearly mirrored in the apoptotic profile of the treated tissues. Ulvan downregulated Bcl-2 expression while up-regulating Bax levels, consequently causing a shift in the Bax/Bcl-2 ratio in favor of mitochondrial outer membrane permeabilization (Sajadimajd et al., 2019). This transition has been assigned a critical checkpoint role within the intrinsic pathway of apoptosis, where there is the release of cytochrome c and activation of the caspase cascade. The concomitant enhancement in caspase-3 activity ensures that ulvan acts as a trigger for a mitochondrial pathway of apoptosis rather than a death pathway (Bhuyan et al., 2023; Wu L. et al., 2025). Notably, this apoptotic induction was not associated with widespread necrosis, as observed in cisplatin-treated tissues, but rather with well-defined apoptotic morphology, including nuclear condensation, chromatin margination, and cellular shrinkage (Henn, 2022). This is a critical difference, given that necrosis typically provokes secondary inflammation and tissue injury, while apoptosis is immunologically silent and biologically controlled; hence, ulvan eradicates tumor cells by programmed death mechanisms rather than through catastrophic destruction (Bhuyan et al., 2023).

Autophagy is a regulative manner, closely related to another pathway, displaying either cytoprotective or cytotoxic properties depending on the signals (Debnath et al., 2023). Results for the expression of genes and the immunohistochemistry results demonstrate the cytoprotective autophagy response initiated by the compound ulvan, which is indicated by the downregulation of mTOR and the upregulation of ATG5 and ATG7 during the maturation step in the cellular process. An enhanced immunohistochemistry staining for the protein LC3 further confirms the formation of autophagosomes. Conversely, the expression does not demonstrate a response mechanism, as the co-activation of apoptosis shows a well-orchestrated response for the death of the cell (Hassa, 2009). In an autophagy-mediated process, the damaged mitochondria and protein aggregation lead to the activation of the caspase response in the mitochondrial permeabilization, making it a very effective anti-tumor cellular response (Rahman et al., 2025; L et al., 2024).

It shows that ulvan-loaded nano-selenium has robust antitumor efficacy because selenium can modulate the intracellular redox and induce apoptosis. The higher anti-cancer efficacy of the ulvan-SeNPs formulation relative to the individual components despite the lower doses of ulvan and selenium delivered, reveals a potent nanoformulation effect, which may be due to enhanced bioavailability, increased cellular uptake and synergistic pathway modulation. Nano-selenium has shown greater production of ROS, which leads to the induction of programmed cell death in cancer cells, evident from the greater expression of Bax, activation of caspase-3, and reduced activity of mTOR. But again, there’s greater stress here, as nano-selenium has disrupted the redox state induced by ulvan, and ulvan could be considered a less stressful approach towards tumorous signaling (Ansari et al., 2024; Gorska et al., 2021). As such, nano-selenium would be more appropriate in short-term tumor debulking applications rather than as part of long-term therapy (Hosnedlova et al., 2018). Histopathological findings gave critical morphological confirmation to these molecular mechanisms. Tissues treated with ulvan manifested reduced tumor burden, with preserved tissue architecture and marked features of apoptosis, characteristic of regulated cell death. In contrast, the cisplatin-treated tissues showed extensive necrosis, hemorrhage, and structural disintegration, indicative of nonspecific toxicity. Tissues treated with nano-selenium showed profound tumor regression with intermediate tissue preservation, reflecting its hybrid mechanism of regulated signaling disruption and oxidative stress–mediated cytotoxicity (Turovsky et al., 2025; Deng et al., 2023).

Collectively, the results indicate that ulvan demonstrates anticancer action through multitargeting involving angiogenic pathways, inflammation, redox imbalance, and apoptosis, as well as autophagy, inducing a systemic change in the tumor biology rather than targeting unconnected pathways. The overall therapeutic context attempts to make the survival of the tumors difficult, and simultaneously, the nanoselenium formulation increases the efficacy at the expense of enhancing cellular stress, suggesting a delicate balance between efficiency and maintaining homeostasis (Zhou and Li, 2022; Li et al., 2025; Wang Q. et al., 2023). The multitargeting pharmacology of ulvan makes it different from the standard chemotherapeutic agents and suggests a promising target for combination cancer therapy targeting long-term biological preservation and diminished toxicity. Mechanistically, ulvan modulates the function of major receptors, such as VEGFR-2, CD44, and TLR-2/4, interrupting tumor development by targeting the upstream as well as downstream regulatory mechanisms, such as NF-κB and mTOR, and hence, inducing a tumorous unfavorable microenvironment with minimal necrosis.

The importance of the study is the distinct combination of ulvan in cancer medicine, as compared to the usual chemotherapeutic agents, which induce large amounts of oxidative stress. The role of ulvan, which preserves histopathological integrity and modulates various pathways of cancer, including apoptosis and angiogenesis, is underlined. The supplement of selenium nanoparticles further advances the anticancer properties of ulvan, inducing apoptosis and regression of cancer, though it also induces stress. The study proposes the combination of ulvan as a therapeutic modality for the management of cancer and ulvan-nano-selenium for higher cytotoxic activity, depending upon the severity of the cancer. The limitations are the requirement for future studies of autophagic flux and the analysis of the immune cells.

## 5 Conclusion

This study presents strong evidence that ulvan, a natural sulfated polysaccharide derived from Ulva lactuca, demonstrates considerable anticancer efficacy in the Ehrlich solid carcinoma model via a well-coordinated, multifaceted mechanism of action. Ulvan exhibited a marked reduction in oxidative stress, suppressed inflammatory signaling, promoted apoptosis, and started a regulatory process of autophagy, contributing to the regression of cancer while preserving normal tissues. The complementation of molecular docking studies with *in vivo* experiments has revealed that ulvan targets multiple molecular targets associated with cancer, and not solely one, thus proving its overall biological activity. Notably, the ulvan-induced autophagic process was regulatory and not uncontrolled, signifying its role in cancer suppression, which works in conjunction with apoptosis, unlike cancer survival. The ulvan-loaded nano-selenium formulation revealed enhanced anticancer efficacy, enhancing apoptotic and autophagic responses while substantially reducing the burden of tumors. However, this enhanced efficacy was linked to elevated cellular stress, suggesting that while nano-selenium enhances therapeutic results, ulvan alone offers a more balanced and homeostatic anticancer profile. In conclusion, ulvan has been unveiled as a promising, natural, and safe anticancer agent with the ability to appropriately modulate all important biological processes involved in tumor growth. The converged nanotechnology using selenium has been discovered as a highly promising strategy that can potently enhance therapeutic efficiency. The current data advocates for more studies using ulvan-based nanotherapies for breast cancer.

## Data Availability

The data supporting the findings of this study are available from the corresponding author upon reasonable request.
